# Comparison of the Regenerative Metabolic Efficiency of Lipid Extracts from Microalgae *Nannochloropsis oceanica* and *Chlorococcum amblystomatis* on Fibroblasts

**DOI:** 10.3390/antiox13030276

**Published:** 2024-02-24

**Authors:** Anna Stasiewicz, Tiago Conde, Maria do Rosario Domingues, Pedro Domingues, Michał Biernacki, Elżbieta Skrzydlewska

**Affiliations:** 1 Department of Analytical Chemistry, Medical University of Bialystok, Kilinskiego 1, 15-069 Bialystok, Poland; anna.stasiewicz@umb.edu.pl (A.S.); michal.biernacki@umb.edu.pl (M.B.); 2 Mass Spectrometry Centre, LAQV-REQUIMTE, Department of Chemistry, University of Aveiro, Santiago University Campus, 3810-193 Aveiro, Portugal; tiagoalexandreconde@ua.pt (T.C.); mrd@ua.pt (M.d.R.D.); p.domingues@ua.pt (P.D.); 3 CESAM—Centre for Environmental and Marine Studies, Department of Chemistry, University of Aveiro, Santiago University Campus, 3810-193 Aveiro, Portugal

**Keywords:** microalgae lipids extracts, fibroblasts, redox balance, inflammation UV radiation, phospholipid and free fatty acids metabolism

## Abstract

UVA radiation leads to oxidative stress and inflammation in skin cells. Therefore, the aim of this study was to compare the effect of lipid extracts from microalgae *Nannochloropsis oceanica* (*N.o.*) (marine) and *Chlorococcum amblystomatis* (*C.a.*) (freshwater) on the redox balance and PUFA metabolism in human skin fibroblasts modified by UVA. Lipid extracts from both types of microalgae introduced into the fibroblast medium after UVA irradiation significantly reduced the level of ROS and enhanced expression of Nrf2, which increased the activity/level of antioxidants (SOD1/2, CAT, GSH, Trx). The reduction in oxidative stress was accompanied by a decrease in the level of 4-HNE, its protein adducts and protein carbonyl groups. Microalgae also reduced the activity of COX1/2, FAAH and MAGL increased by UVA, and as a consequence, the level of lipid mediators (especially after *N.o.*) decreased, both from the group of endocannabinoids (AEA, 2-AG, PEA) and eicosanoids (PGE2, 15d-PGJ2, TXB2, 15-HETE), acting mainly through receptors related to G protein, the expression of which increases after UVA. This further contributed to the reduction in oxidative stress and pro-inflammatory signaling at NF-κB and TNFα levels. Therefore, it is suggested that lipid extracts from both *N.o.* and *C.a.* microalgae can be used to regenerate fibroblast metabolism disturbed by UVA radiation.

## 1. Introduction

Skin is the major barrier to protect the human body against the negative effects of physicochemical and biological external factors, including UV radiation contained in sunlight, which is one of the main factors of physical stress [1]. The light UV spectrum includes UVA (320–400 nm) and UVB (280–320 nm) components, which have different capabilities of reaching layers of the skin and generate different biological effects [2]. However, both UVA and UVB radiation are particularly useful in the treatment of skin diseases such as eczema and psoriasis [3]. It is known that UVA and UVB radiation have different effects on human skin because UVA radiation both penetrates the dermis as well as reaching deeply to the dermis and small amounts can also reach deeper, while UVB radiation mainly affects the epidermis [2]. UVB radiation primarily penetrates the epidermis, while UVA radiation reaches the dermis, whose basic building cells are fibroblasts, and it is mainly responsible for photoaging, pigmentation and immunomodulation [4]. The cellular targets of UVA radiation include skin chromophores, such as urocanic acid, riboflavin porphyrins and melanin, which are involved in the production of ROS [5]. UVA also releases factors that catalyze the generation of ROS, such as free iron ions and heme [6]. As a consequence, UVA radiation can disturb the redox homeostasis within the cells, favoring oxidative modifications of cell components, including proteins and lipids, such as membrane phospholipids [7]. Thus, UVA radiation has consequences at transcription factor levels, such as Nrf2, which is responsible for cellular antioxidant response and anti-inflammatory and pro-inflammatory cytokines, as well as a transcription factor regulating pro-inflammatory signaling (NF-κB) and adhesion molecules [8,9]. However, changes in the structure of phospholipids include the result of the direct interaction of ROS with polyunsaturated fatty acids (PUFAs), which are an integral component of phospholipid structures, which leads to the generation of lipid peroxidation products, such as 4-hydroxynonenal and prostaglandin derivatives (neuroprostanes and isoprostanes), among others. Ceramides and oxidized phospholipids participate, e.g., in the modulation of the inflammatory response [10,11]. Moreover, as a result of the activation of phospholipid-metabolizing enzymes, such as phospholipases (PLAs), which release PUFAs from phospholipid structures [12,13], further ROS-dependent lipid peroxidation occurs with the generation of small-molecule, electrophilic aldehydes that can further modify the structures of other macromolecular compounds, including proteins and nucleic acids [14], reducing their biological effectiveness [15]. However, PLAs, COXs and LOXs, acting on free PUFAs, intensify the generation of lipid mediators, including endocannabinoids and eicosanoids, whose biological activity directly and indirectly, through the activation of membrane receptors, mainly G-protein-coupled receptors, modifies both oxidative stress (by modifying the generation of ROS) and inflammatory signaling (by modifying the generation of pro-inflammatory factors, e.g., TNFα) [16,17]. Therefore, modifications of phospholipid metabolism additionally promote modifications of the structure and function of cellular proteins, which affects cellular metabolism [18].

Taking into account the daily exposure of skin cells to the consequences of oxidative stress also includes changes in the composition and structure of membrane phospholipids, which changes the permeability of membranes to both endo- and exogenous compounds and thus modifies the functioning of the cell and intercellular signaling [19,20,21,22]. For UV radiation, this situation forces the search for protective and regenerative compounds, especially among those of natural origin, which generally generate fewer side effects. One of the sources of such compounds is marine microalgae, which are photosynthetic organisms that generate many metabolically important compounds, including proteins, lipids, fatty acids and carbohydrates, but also carotenoids [23]. Due to the ingredients they contain, special attention is paid to the following species of algae from both marine and freshwater sources, such as the following: *Chlorella vulgaris*, *Chlorococcum amblystomatis*, *Nannochloropsis oceanica*, *Phaeodactylum tricornutum*, *Scenedesmus obliquus* and *Spirulina* sp. [24,25].

Recently, it has been shown that the freshwater microalgae *Chlorococcum amblystomatis* (*C.a.*) is an excellent source of fatty acids belonging to the ω-3 and ω-6, including α-linolenic acid (ALA; C18:3 (n-3)), the content of which ranges from 19.4% up to 31.4% of the total fatty acid content [26,27]. In addition, these microalgae contain eicosapentaenoic acid (EPA; C20:5 (n-3)) and palmitoleic acid (POA; C16:1Δ9 (n-7)) [24,27], which, through limiting the expression of pro-inflammatory factors, have anti-inflammatory effects, and by modulating lipid metabolism, they influence the fluidity of biological membranes [28,29]. However, phosphatidylinositols containing palmitoleic acid in their structure act as mitogens, significantly activating the proliferation of fibroblasts [29]. In addition to these fatty acids, the microalgae *C.a.* is characterized by a high content of chlorophyll α and β [30], which allows the production of preparations supporting the treatment of cardiovascular diseases and complications [31]. Additionally, it has been shown that chlorophyll, in cooperation with lutein, also presents in this microalgae; these are strong antioxidants which additionally feed the body, detoxify the body, and also support executive units and maintain the hormonal balance of the human body by binding and removing certain endocrine-disrupting substances [27,30,31].

The second (marine) microalgae used to assess the therapeutic use is *Nannochloropsis oceanica* (*N.o.*), which is a rich source of eicosapentaenoic acid (approx. 30% of all fatty acids contained). Due to the involvement of EPA in supporting the metabolism of lipids and sugars, and its anti-inflammatory and neuroprotective effects [32,33], this microalga is increasingly used in the pharmaceutical and nutraceutical industries [24,34]. Moreover, *N.o.* microalgae, in a similar amount to *C.a.*, contains palmitoleic acid (POA) [24,35], which has anti-inflammatory properties [28]. Moreover, *N.o.* microalgae are a rich source of endogenous amino acids such as glutamyl acid, proline and aspartic acid, which are neurotransmitters, immunomodulators and factors involved in the regulation of the body’s hormonal balance [36,37,38], but it also contains exogenous amino acids such as lysine, leucine, threonine and phenylalanine, which are necessary for proper protein biosynthesis [39].

Consequently, this study compared the regenerative effect of lipid extracts obtained from the marine microalgae *N.o.* and the freshwater microalgae *C.a* on redox balance and pro-inflammatory signaling, including the direct and indirect participation of cellular phospholipid metabolites in fibroblasts irradiated with UVA.

## 2. Materials and Methods

### 2.1. Materials and Cell Culture Treatment

#### 2.1.1. Microalgaes

Biomasses of spray-dried marine microalgae *Nannochloropsis oceanica* and freshwater microalgae *Chlorococcum amblystomatis* obtained from companies producing them in Portugal (see below) were used for research.

The biomass of the spray-dried microalgae *Nannochloropsis oceanica* was provided by Allmicroalgae, Natural products S.A., located in Rua 25 de Abril s/n 2445-413 Pataias, Portugal. The microalgae were cultured in Guillard F2 medium adapted to local water [40]. It was supplemented with a mixture of magnesium (Necton, Olhão, Portugal) and NaCl (Salexpor, Coimbra, Portugal) with a salinity of 30 gL^−1^. *Nannochloropsis oceanica* was grown in 5 L flask reactors with continuous light exposure of 700 μmol photons m^2^s^−1^ from 7 to 15 days. The inoculation of one outdoor reactor holding 0.1 m^3^ with a flat panel (FP) required the use of five 5 L flask reactors, after which it was scaled up to 1 m^3^. Four FPs were used to inoculate the culture in a 10 m^3^ tubular photobioreactor (FBR). After the inoculation process, the reactor was exposed to light and ambient temperature until stable parameters were achieved. A sprinkler irrigation system was used to prevent overheating of the PBR reactor, while a stable ambient pH was achieved by pulsed carbon dioxide dosing. Portions containing about 50 g of microalgae in a volume of 1 L were subjected to drying in a spray dryer (dryer operating efficiency of 150 kg of H_2_O per hour) using an air stream at 215 ± 5 °C, which allowed rapid drying of the material. The dried algae were subjected to grinding in a cyclone, and the powdered algae were stored until the determinations were made in a place without access to light and moisture.

The biomass of the spray-dried microalgae *Chlorococcum amblystomatis* was provided by Allmicroalgae, Natural products S.A., (Fábrica Cibra Pataias, 2445-287 Pataias, Portugal). The microalgae were cultured in Guillard F2 medium adapted to local water supplemented with nitrates (nitrogen source). For the algae *Chlorococcum amblystomatis*, an analogous culture method and conditions were used as for the algae *Nannochloropsis oceanica*. The microalgae were grown in 5 L flask reactors with continuous exposure to light from 7 to 15 days. The inoculation of one outdoor reactor holding 0.1 m^3^ with a flat panel (FP) required the use of five 5 L flask reactors. The *C.a.* microalgae were scaled up gradually, starting with 0.1 m^3^ to 0.25 m^3^, then to 0.5 m^3^, ending with a 1 m^3^ flat panel (FP) reactor. Four FPs were used to inoculate the culture in a 10 m^3^ tubular photobioreactor (FBR). After the inoculation process, the reactor was exposed to environmental factors (average temperature of 15.5 °C and light irradiance of 20.10 MJ·m^−2^·day^−1^) for 21 days. A sprinkler irrigation system was used to prevent the temperature rising over 28 °C in the PBR reactor, while the constant pH 7.0–8.0 was achieved by pulsed carbon dioxide dosing. Portions of microalgae were subjected to drying in a spray dryer (dryer operating efficiency of 150 kg of H_2_O per hour) using an air stream at 215 ± 5 °C, which allowed rapid drying of the material. The dried algae were subjected to grinding in a cyclone, and the powdered algae were stored until the determinations were made in a place without access to light and moisture.

#### 2.1.2. Microalgae Lipid Extracts

The lipid extracts of *N.o.* and *C.a.* were obtained by the previously described Folch method [41]. An extraction mixture consisting of dichloromethane and methanol (2:1, *v*/*v*) was added to 25 mg of microalgae and then processed by four cycles of stirring and centrifugation (670× *g* for 10 min). The supernatants obtained in this process were evaporated to dryness using nitrogen, and the dry residue was then suspended in a dichloromethane:methanol mixture and subjected to centrifugation, after which Mili-Q water was added to the samples. After centrifugation under the previously described conditions, phase separation was achieved, after which the aqueous phase was extracted two more times. The collected and combined organic phases separated from the microalgae were then subjected to gravimetric analysis to determine lipid content.

#### 2.1.3. Phospholipid and Glycolipid Quantification

The phospholipid (PL) content in the lipid extracts was determined according to the Bartlett and Lewis phosphorus measurement methodology [42]. Sample aliquots were transferred to glass tubes and dried under a nitrogen stream and phosphate standards were prepared from a solution of NaH_2_PO_4_·2H_2_O (100 μg·mL^−1^ of P). A solution of 70% perchloric acid was added to samples and heated for 1 h at 180 °C in a heating block (Stuart, Staffordshire, UK). This solution was also added to the standards. Then, Milli-Q water was added, followed by the addition of a 2.5% ammonium molybdate solution. Both samples and standards were vortexed well, and a solution of 10% ascorbic acid was added, followed by incubation for 10 min at 100 °C in a water bath and cooling after at room temperature. The absorbance was measured at 797 nm in an ultraviolet-visible (UV–Vis) spectrophotometer (Multiskan GO, Thermo Scientific, Hudson, NH, USA). Two duplicates of two independent measurements were carried out for each sample. The amount of PL was calculated by multiplying the quantity of determined phosphorus (μg) by the PL conversion factor value of 25 [43].

The glycolipid (GL) content in the lipid extracts was estimated through the orcinol assay, according to Cyberlipids [44], and was determined through the hexose content (% of glucose). Sample aliquots were added to glass tubes and dried under nitrogen stream. D-glucose solution (2 mg·mL^−1^) was used to prepare different standards for a calibration curve. An orcinol solution in 70% of sulfuric acid was added to both samples and standards, followed by incubation for 20 min at 80 °C in a heating block (Stuart, Staffordshire, UK). Then, the absorbance was measured at 505 nm using a UV–Vis spectrophotometer (Multiskan GO, Thermo Scientific, Hudson, NH, USA). Two duplicates of two independent measurements were performed for each sample and standard. The amount of GL was calculated by multiplying the quantity of determined sugar by the conversion factor of 2.8 [45].

The data obtained for the determination of phospholipids and glycolipids in the lipid extracts from *Nannochloropsis oceanica* and *Chlorococcum amblystomatis* were collected and are presented in Supplementary Table S1.

#### 2.1.4. Lipid Profiles of *Nannochloropsis oceanica* and *Chlorococcum amblystomatis* Extracts Analysis

Hydrophilic interaction liquid chromatography coupled to high-resolution mass spectrometry (HILIC-MS) as well as tandem mass spectrometry (MS/MS) using a Q-Exactive Orbitrap hybrid quadrupole mass spectrometer (Thermo Fisher Scientific, Bremen, Germany) were used to analyze the lipid profiles of the extracts obtained from *N.o.* and *C.a.*, as previously described [35]. A total of 40 μg of the resulting lipid extracts were suspended in an eluent aliquot consisting of 95% mobile phase B (CH_3_CN/CH_3_OH (60:40, *v*/*v*) with 5 mM ammonium acetate) and 5% mobile phase A (H_2_O/CH_3_CN/CH_3_OH (25:50:25, *v*/*v*/*v*) with 5 mM ammonium acetate) and a mixture of lipid standard solutions (dMPC-0.04 μg, LPC-0.04 μg, dMPE-0.04 μg, dMPG-0.024 μg and Cer(17:0/d18:1)-0.04 μg). Five μL of the resulting mixture was injected onto a low-porosity Ascentis^®^ Express column (10 cm × 2.1 mm, 2.7 μm; Sigma-Aldrich, St. Louis, MO, USA). The analysis was carried out at 35 °C using a flow rate of 200 μL·min^−1^ and the following gradient elution: in the initial phase (0–2 min), the proportion of phase B was 95%, followed by a linear decrease in the proportion of phase B to 52% (2–10 min), and then to 35% (10–15 min), followed successively by stabilization of the elution (15–17 min), a return to the initial gradient conditions (17–20 min) and stabilization of the column conditions (20–30 min). Simultaneously positive (ESI 3.0 kV) and negative (ESI −2.7 kV) pacing modes of the mass spectrometer with automatic gain control (AGC) 1 × 106 and high resolution (70,000) were set. The gas flow rate in the sheath was 20U, and the capillary temperature was 250 °C. MS/MS determinations were performed at a collision energy™(CE) between 25, 30, 35 eV and a resolution of 17,500. Data collection was performed using the Xcalibur data system (V3.3, Thermo Fisher Scientific, Waltham, MA, USA). Identification of molecular species of polar lipids was based on LC retention time, mass accuracy (<5 ppm) and detailed structural information inferred from MS/MS data [35]. Supplementary Tables S2 and S3 show the collected data forming the lipid profile of extracts extracted from *Nannochloropsis oceanica* and *Chlorococcum amblystomatis*.

#### 2.1.5. Cell Culture

The study of the effect of lipid extracts from *Nannochloropsis oceanica* and *Chlorococcum amblystomatis* was carried out using human skin fibroblast cells (CRL-1474) obtained from the American Type Culture Collection (ATCC). Sterile reagents necessary for cell culture were purchased from Thermo Fisher Scientific (Waltham, MA, USA), while other reagents were obtained from Sigma-Aldrich Chemical Co. (St. Louis, MO, USA). LC-MS-grade solvents were used, and the water used in the determinations was Milli-Q water, obtained using the Milli-Q Millipore system (Advantage A10, Millipore Corporation, Billerica, MA, USA) with a filter with pore diameter of 0.22 µm.

Cell culture was conducted in a two-dimensional model at 37 °C in a humidified atmosphere of 5% CO_2_ using Dulbecco’s Modified Eagle Medium (DMEM) enriched with fetal bovine serum (FBS; 10%) and streptomycin (50 μg/mL) and penicillin (50 U/mL). After reaching 70% cofluency, passage 11 cells were washed with phosphate-buffered saline (PBS) and in this cold buffer (4 °C) subjected to UVA irradiation (365 nm; dose 20 J/cm^2^) using a Bio-Link Crosslinker BLX 365/312 (Vilber Lourmat, Germany). A previous report has shown [46] that under such conditions, fibroblast metabolism assessed by the MTT (3-(4,5-dimethylthiazol-2-yl)-2,5-diphenyltetrazolium bromide) method suggests an estimated cell survival rate of 70%. Under such conditions, there is increased activation of pro-oxidant mechanisms in cells [47]. The process of irradiating the cells was carried out maintaining a constant distance between the plates and the lamps of 15 cm; additionally, cold PBS (phosphate-buffered saline, 4 °C) was used to reduce the risk of heat stress.

After completing the cell irradiation process and changing the mediums to new ones, fibroblasts were treated with the tested extracts from the microalgae *C.a.* and *N.o.* in 0.1% dimethyl sulfoxide (DMSO) at concentrations from 1 to 100 µg/mL. The cells remained exposed to the extracts for 24 h under standard cell culture conditions. In parallel, fibroblast cultures were carried out using the *N.o*. and *C.a.* extracts in 0.1% DMSO. Based on the results obtained in the MTT test and the lack of observed changes in the viability of the fibroblasts (compared to the control) for both types of microalgae, a concentration of 2 µg/mL was selected to conduct the experiments [47]. After 24 h incubation, the cells were washed with cold PBS and then scraped on ice. The collected cells were successively disintegrated and centrifuged, and the obtained cell lysates were used for examinations. The total protein content in cell lysates was determined using the Bradford test [48].

### 2.2. Methods

#### 2.2.1. Determination of ROS Generation

Superoxide anion generation was measured by evaluating the formation of stable CM nitroxide radicals analyzed with an EPR spectrometer (Noxygen GmbH/Bruker Biospin GmbH, Ettlingen, Germany) [49]. The results are presented as micromoles of O_2_^−^ generated per minute and normalized per milligram of protein.

#### 2.2.2. Antioxidant Enzyme Activity and Low-Molecular Antioxidant Level Estimation

The activity of cytosolic, cuprum and zincum-dependent superoxide dismutase-SOD1 (EC.1.15.1.1) and mitochondrial, manganese-dependent superoxide dismutase-SOD2 (EC.1.15.1.1) was quantified spectrophotometrically with a wavelength of 480 nm [50,51]. The final result was obtained by comparing the obtained value with the control in which carbonate buffer containing 100 µM EDTA with pH = 10.2 was used instead of the sample. The activities of both enzymes were calculated and the results obtained for both enzymes are presented in U/mg of protein.

The enzymatic activity of catalase (CAT—EC.1.11.1.9) was tested by the spectrophotometric method based on the reaction efficiency of the decomposition of hydrogen peroxide (H_2_O_2_) by catalase [52], measuring the absorption at a wavelength of 240 nm. One unit of CAT activity was determined on the basis of the amount of enzyme catalyzing the degradation of H_2_O_2_ (1 μmol) into H_2_O and O_2_ within 1 min. The obtained results were normalized by converting them to the protein concentration in the samples, and the final values are expressed as U/mg protein.

Glutathione peroxidase (GSHPx–EC.1.11.1.6) activity was estimated based on oxidized glutathione (GSSG) reduction with simultaneous NADPH oxidation, resulting in reduced absorption of radiation at a wavelength of 340 nm [53]. One unit of GSHPx activity was determined on the basis of the amount of enzyme oxidizing equivalent of 1 µmol of NADPH within 1 min at room temperature and an environmental pH of 7.4. The obtained results were normalized by the protein concentrations in the samples, and the final unit is expressed as mU/mg protein.

The enzymatic activity of glutathione reductase (GSSG-R-EC.1.6.4.2) was estimated based on oxidized glutathione (GSSG) reduction with simultaneous NADPH oxidation, resulting in reduced absorption of radiation at a wavelength of 340 nm [54]. One unit of GSSGR activity was determined on the basis of the amount of enzyme oxidizing 1 µmol of NADPH within 1 min compared to 0.02 M phosphate buffer control (pH 7.0). The obtained results were normalized by the protein concentrations in the samples, and the final unit is expressed as mU/mg protein.

The amount of reduced glutathione (GSH) was measured using capillary electrophoresis (CE) [55] with a 47 cm long capillary working at 27 kV. UV detection was used at a wavelength of 200 nm. The GSH level was determined based on a calibration curve prepared in the range from 1 to 120 nmol/mL (r^2^ −0.9984). The obtained results were normalized by the protein concentrations in the samples, and the final unit is expressed as nmol/mg protein.

The enzymatic activity of thioredoxin reductase (TrxR-EC.1.8.1.9) was assessed with a commercially available kit from Sigma-Aldrich (St. Louis, MO, USA). This test is based on the reduction in 2-nitrobenzoic acid (DTNB) to 5-thio-2-nitrobenzoic acid (TNB) by NADPH [56], which results in an increase in absorbance at 412 nm. The unit of TrxR activity is expressed as the amount of TrxR which, at a temperature of 25 °C and an environmental pH of 7.0, causes an increase in radiation absorbance per 1 mL of the tested sample. The obtained results were normalized by the protein concentrations in the samples, resulting in the final unit expressed as U/mg protein.

The thioredoxin (Trx) level was determined by ELISA (enzymatic immunoassay) [56]. To measure Trx levels, a primary thioredoxin antibody (Abcam, Cambridge, MA, USA) was applied to the plates with the test samples, followed by incubation at 4 °C for 24 h. In the next step, the antibody was removed and blocking solution peroxidase activity was applied (PBS enriched with hydrogen peroxide at a concentration of 3%) to the plates, and then the blocking solution was removed and a secondary goat anti-rabbit antibody was applied to the plate (Dako, Carpinteria, CA, USA) and incubated for 1 h. After this time, the secondary antibody was removed, and a chromogen solution containing 0.1 mg/mL 3,3′,5,5′-Tetramethylbenzidine (TMB) with 0.012% hydrogen peroxide was introduced into the samples. The addition of 2 M sulfuric acid stopped the reaction. The amount of thioredoxin was estimated based on the absorption of radiation with a wavelength of 450 nm, using an additional 620 nm as a reference wavelength. The obtained results were calculated based on the calibration curve and normalized by the protein concentrations in the samples, resulting in the final unit expressed as µg/mg protein.

#### 2.2.3. Protein Level

The levels of the selected proteins NF-κB p65, (OriGene, Rockville, MD, USA); TNFα (Merck, Darmstadt, Germany), NF-κB p52 (LSBio, Seattle, WA, USA), HO-1 (Enzo Life Sciences, Farmingdale, NY, USA), Keap1 (Sino Biological Europe, Düsseldorfer, Eschborn, Germany) and Nrf2 (Gentaur BV, Kampenhout, Belgium) were assessed using the ELISA method [57]. Cell lysates were placed on dedicated ELISA plates (Nunc Immuno Maxi Sorp, Thermo Scientific, Waltham, MA, USA) and subjected to a 3 h incubation at 4 °C with a blocking solution consisting of a carbonate-binding buffer and skimmed milk with a concentration of 5%. After incubation, the samples were washed with a mixture of phosphate-buffered saline containing Tween 20 at a concentration of 0.1% (PBS-T), and then the appropriate primary antibodies (host: mouse) were applied: Trx, NF-κB (p65), NF-κB (p52), TNFα (Sigma-Aldrich, St. Louis, MO, USA), HO-1 (Invitrogen, Walthman, MA, USA), Nrf2 (Sigma-Aldrich, St. Louis, MO, USA) and Keap1 (Sigma-Aldrich, St. Louis, MO, USA). A 1:1000 dilution was used for each antibody. Incubation with primary antibodies was carried out for 24 h at 4 °C, after which the samples were washed with a TBS-T mixture, and then blocking solution peroxidase activity was applied (PBS enriched with hydrogen peroxide at a concentration of 3% and skimmed milk powder at a concentration of 3%), and they were incubated at room temperature. After removal of the blocking solution, goat solution anti-rabbit/mouse EnVision+ Dual Link/HRP (1:100) (Agilent Technologies, Santa Clara, CA, USA) as the secondary antibody was added and incubated at room temperature for 1 h. After this time, the secondary antibody was removed, and a chromogen solution containing 0.1 mg/mL 3,3′,5,5′-Tetramethylbenzidine (TMB) with 0.012% hydrogen peroxide was added to the samples and incubated for 40 min. The addition of 2 M sulfuric acid stopped the reaction, and 10 min after the end of the reaction, a spectrophotometric measurement was carried out at a wavelength of 450 nm. The obtained results were calculated based on calibration curves for each protein (1–5 µg/mg prot. for NF-κB p65; 1–5 µg/mg prot. for TNFα; 1–5 µg/mg prot. for NF-κB p52; 1–5 µg/mg prot. for HO-1; 1–5 µg/mg prot. for Keap1; 1–5 µg/mg prot. for Nrf2).

#### 2.2.4. Lipid Peroxidation Product (4-HNE) Determination

The level of 4-hydroxynonenal (4-HNE) as a representative small molecule lipid peroxidation product was determined using gas chromatography coupled with mass spectrometer in selected ion monitoring (SIM) mode based on the Tsikas method [58] with minor modifications as described previously [59] and with 4-hydroxynonenal-d_3_ as an internal standard (ISTD). The first stage of the analysis included adding the internal standard and the PFBHA·HCl derivatization reagent solution to the sample and incubating them on a shaker at room temperature for 24 h. The derivatization process was interrupted by the addition of methanol, and then the aldehydes were extracted into hexane, after which the samples were centrifuged and the hexane layer was collected in glass tubes with a modified, chemically inactive surface. The samples were re-extracted and the hexane layers were combined and then evaporated to dryness. The dry residue was dissolved in a portion of the second derivatization reagent (BSTFA:TMCS) and incubated at 80 °C for 15 min. The samples were mixed, cooled and then transferred to chromatographic vials with glass inserts with a modified, chemically inactive surface; O-PFB-oxime-TMS derivatives were analyzed for both 4-HNE and ISTD using 7890A GC—7000 quadrupole MS/MS (Agilent Technologies, Palo Alto, CA, USA) equipped with a HP-5MS capillary column (30-m length, 0.25-mm internal diameter). The analysis of 4-HNE content was performed based on ions *m*/*z* 242.0 and *m*/*z* 245.0 for 4-HNE-PFB-TMS and the ISTD derivative, respectively. The 4-HNE values obtained from the analysis were normalized by the protein concentrations in the samples, and the final unit expressed is as nmol/mg protein.

#### 2.2.5. Adducts of 4-HNE—Protein Determination

Modifications of fibroblast protein structure resulting from oxidative conditions were assessed based on the tendency to form adducts between 4-HNE and proteins present in cells measured by the ELISA method described in Section 2.2.3 [57]. In this assay, a monoclonal mouse anti-4-HNE-protein antibody (Invitrogen, Burlington, ON, Canada) and goat anti-rabbit/mouse EnVision+ Dual Link/HRP (1:100) (Agilent Technologies, Santa Clara, CA, USA) were used as primary and secondary antibodies, respectively. To determine the amount of formed adducts, a calibration curve was prepared in the concentration range of 0.5 to 25 pmol/mg BSE (r^2^ −0.9987), and the calculated results were normalized by the protein concentrations in the samples, resulting in the final unit expressed as pg/mg protein.

#### 2.2.6. Protein Carbonyl Group (CBO) Determination

One of the parameters used to assess the intensity of oxidative processes causing modifications in proteins is the level of protein carbonyl groups (CBOs). This indicator is assessed based on the reaction of 2,4-dinitrophenylhydrazine (DNPH) and the carbonyl groups of oxidized proteins [60], which results in the production of hydrazone. The concentration of the product formed in the reaction is determined by measuring the absorption of radiation with a wavelength of 370 nm. The obtained results are expressed in concentration units and normalized by the protein concentrations in the samples, resulting in the final unit expressed as nmol/mg protein.

#### 2.2.7. Determination of the Activity of Enzyme-Metabolizing Phospholipids and Free PUFAs

The activities of enzymes involved in phospholipid metabolism (phospholipase A2 (cPLA2-EC.3.1.1.4); cyclooxygenases 1 and 2 (COX-1/2-EC.1.14.99.1); lipoxygenase 5 (LOX-5-EC1.13.11.58)) were examined spectrophotometrically using commercially available kits. These assays were carried out according to the developer’s instructions as follows: PLA2 Assay Kit (Cayman Chemical Company, Ann Arbor, MI, USA), COX-1/2 commercial assay kit (Cayman Chemical Company, Ann Arbor, MI, USA), LOX-5 commercial assay kit (Sigma-Aldrich, Steinheim, Germany).

The arachidonoyl thio-PC was applied to obtain PLA2 activity, as the enzyme hydrolyzes the sn-2 position bond of the substrate, thus releasing free thiol as a product. This thiol in turn reacts with (5,5′-dithio-bis(2-nitrobenzoic) acid (DTNB) to give the product, which is determined spectrophotometrically using a wavelength of 405 nm [61]. One unit expresses the amount of arachidonoyl Thio-PC (in nmol) hydrolyzed by one µmol of enzyme during one minute at room temperature. The obtained results were normalized by taking into account the protein concentrations in the samples.

The enzymatic activities of COX 1 and 2 were determined by a reaction involving the oxidation of N,N,N′,N′-tetramethyl-p-phenylenediamine (TMPD) [62]. The resulting product was determined spectrophotometrically at 590 nm, using SC-560 as an inhibitor of COX-1 activity (inhibitor included in the kit) to determine COX2 activity. The activity of both cyclooxygenases is presented as a unit (U) equivalent to the amount of TMPD formed (in nmol) in 1 min. The obtained results were normalized by taking into account the protein concentrations in the samples.

The enzymatic activity of lipoxygenase-5 (LOX-5) was measured by the reaction of the LOX substrate converted into an intermediate compound that reacts with the LOX probe, generating a fluorescent product. The increase in fluorescence proportional to LOX-5 activity was checked under λ_Ex_ = 500 nm and λ_Em_ = 536 nm conditions and expressed in mU/mg protein, where one unit (U) is equivalent to the amount of enzyme, leading to the oxidation of 1 μmol of LOX probe during 1 min at 25 °C and an environmental pH of 7.4.

#### 2.2.8. Phospholipid and Free Fatty Acid Determination

Phospholipids and free fatty acids were isolated by the Folch method using a solution of CHCl_3_/CH_3_OH (2:1, *v*/*v*) with butylated hydroxytoluene (0,01%) [63]. Fatty acid (FA) fractions were separated using thin layer chromatography (TLC) analysis with a solution containing heptane-diisopropyl ether–acetic acid (60:40:3, *v*/*v*/*v*) as an eluent. Boron trifluoride in methanol was used to carry out the methylation process for both FA fractions to obtain fatty acid methyl esters (FAMEs). Fatty acid content was obtained by gas chromatography with a flame ionization detector (FID) on Clarus 500 Gas Chromatograph (Perkin Elmer) with capillary column coated with Varian CP-Sil88 stationary phase (50 m × 0.25 mm, ID 0.2 μm, Varian). Qualitative and quantitative analysis of FAMEs was carried out by comparing their retention times with standards and using an internal standard (ISTD), respectively. Nonadecanoic acid (19:0) and 1,2-dinonadecanoyl-sn-glycero-3-phosphocholine (19:0 PC) were used as ISTDs for free fatty acid methyl esthers and phospholipid fatty acid methyl esters, respectively. Plasma levels of arachidonic acid (AA), eicosapentaenoic acid (EPA) and docosahexaenoic acid (DHA) in both fractions were obtained and recalculated into μg/mg protein.

#### 2.2.9. Determination of the Level of Eicosanoids

The levels of selected eicosanoids (prostaglandins E2, PGE2; 15-deoxy-delta-12,14-prostaglandin J2, 15-d-PGJ2; thromboxane B2, TXB2; 15-Hydroxyeicosatetraenoic acid, 15-HETE) were analyzed using Nexera X2 Ultra-Performance Liquid Chromatography system with electrospray ionization (ESI) running on negative ion mode using Shimadzu 8060 Triple Quadrupole system (Shimadzu, Kyoto, Japan) [64]. The separation of analytes was achieved at 50 °C using analytical column Eclipse Plus C18 (2.1 × 100 mm, 1.8 µm particle size) and an injection volume of 3 µL. The method used 0.1% acetic acid in Milli-Q water and acetonitrile as mobile phases A and B. A gradient program was used in elution as follows: in the initial phase, the proportion of phase B was 25%, followed by a linear increase in the proportion of phase B to 40% (0–1 min), and then a linear increase in the proportion of phase B changed to 42% (1–2.5 min). In the next phase, a linear increase in phase B to 50% (2.5–4.5 min) was applied, followed by an increase in the phase B proportion to 65% (4.5 to 10.5 min), after which phase B was increased to 75% (10.5–12.5 min) and to 85% (12.5–14.0 min) and ending with a 95% increase in phase B (14.0–14.5 min). The next step of the applied gradient program, after obtaining the highest proportion of the B phase, was the return to the initial gradient conditions (14.5–15.0 min) and the stabilization of the column conditions (15.0–16.0 min). The following fragmentation transitions were selected for eicosanoids analysis: *m*/*z* 351.3→271.2, 315.2→271.2, *m*/*z* 369.3→169.1 and *m*/*z* 319.0→301.2 for PGE2, 15-d-PGJ2, TXB2 and 15-HETE, respectively. The transitions *m*/*z* 355.0→275.3, *m*/*z* 319.3→275.2, 373.0→173.1 and 327.0→226.2 were selected for PGD2-d_4_, 15-d-PGJ2-d_4_, TXB2-d_4_ and 15-HETE-d_8_, which acted as ISTDs. Concentrations of tested eicosanoids are expressed in pmol/mg protein.

#### 2.2.10. Determination of the Level of Endocannabinoids and Its Degrading Enzymes

Quantitative analysis of selected endocannabinoids such as arachidonoyl ethanolamide (AEA) and 2-arachidonylglycerol (2-AG) compounds related to AEA and 2-AG (palmitoylethanolamide, PEA; oleoylethanolamide, OEA) was carried out using a Nexera X2 Ultra-Performance Liquid Chromatography system with electrospray ionization (ESI) running on positive ion mode using a Shimadzu 8060 Triple Quadrupole system (Shimadzu, Kyoto, Japan) [65]. The separation of analytes was achieved by analytical column 120 EC-C18 (3.0 × 150 mm; 2.7 µm particle size) and an injection volume of 5 µL. The method used mobile phases A and B consisting of a solution containing 0.1% formic acid in MilliQ water and acetonitrile, respectively. A gradient program was used in elution as follows: in the initial phase, the proportion of phase B was 70%, followed by a linear increase in the proportion of phase B to 80% (0–5 min), and then a linear increase in the proportion of phase B changed to 88% (5–10 min). In the next phase, a linear increase in phase B to 100% (10–16 min) was applied, followed successively by the stabilization of the elution (16–20 min), a return to the initial gradient conditions (20–21 min) and the stabilization of the column conditions (21–25 min). In the initial stage of sample preparation, freeze-thawing on ice was used, followed by the addition of an internal standard (ISTD) mixture in a volume of 10 µL. The ISTD mixture contained AEA-d_8_, 2-AG-d_8_ and OEA-d_4_ at a concentration of 100 ng/mL for each. After the addition of the ISTD mixture, solid-phase extraction (SPE) of the samples was applied using properly prepared cartridges, which were rinsed and vacuum-dried and eluted after sample loading. The solutions obtained after the elution of analytes were evaporated to dryness, and the resulting dry residue was then dissolved in a mixture of acetonitrile and water (7:3) with 0.1% formic acid. Samples obtained by the procedure described above were transferred to chromatography vials with inserts and underwent LC-MS analysis immediately. The following fragmentation transitions were selected for endocannabinoids analysis: *m*/*z* 348.3→62.15, *m*/*z* 379.3→287.25, *m*/*z* 300.3→62.00 and *m*/*z* 326.0→62.00 for AEA, 2-AG, PEA and OEA, respectively. The transitions *m*/*z* 356.2→63.05, *m*/*z* 387.3→294.0 and *m*/*z* 330.20→66.15 were selected for AEA-d8, 2-AG-d8 and OEA-d4, respectively, which were added as ISTDs. The concentrations of tested endocannabinoids are expressed in fmoles/mg protein.

Changes in endocannabinoid metabolism, being a consequence of the enzymatic activity of the fatty acid amide hydrolase (FAAH-EC-3.5.1.99) and monoacylglycerol lipase (MAGL-EC-3.1.1.23), were assessed using spectrophotometric methods. FAAH enzymatic activity was measured in cells lysed in 20 mM Hepes buffer (pH 7.8) containing 150 mM NaCl, 10% glycerol and 1% Triton X-100 at 4 °C. The samples were centrifuged, and a reaction buffer (125 nm Tris (pH 9.0) + 1 mM EDTA + 1 μM FAAH substrate (decanoyl-m-nitroaniline (m-NA)) was added to obtained supernatant in a ratio of 20:175 μL (supernatant:reaction buffer). The level of m-NA released from the FAAH substrate was measured spectrophotometrically at a wavelength of 410 nm [66] and stated as nmol/min/mg protein. The activity of MAGL was determined in cells lysed in 20 mM Tris buffer containing 320 nM sucrose and 1 mM EDTA (pH 8.0). The samples were centrifuged and the cell supernatants were pre-incubated at 4 °C with 10 mM Tris buffer with 1 mM EDTA (pH 7.2). After 15 min, the samples were treated with arachidonoyl-1-thio-glycerol (A-1-TG) mixture for 5 min in 37 °C. Incubation was followed by cooling and adding 5,5′-dithio-bis (2-nitrobenzoic acid) (DTNB) with a concentration of 1 mM. After 3 min of hydrolysis, the level of 5′-thio-2-nitrobenzoic acid (TNB) released from DTNB was measured at 412 nm [67]. MAGL activity is expressed as nmol/min/mg protein.

#### 2.2.11. Determination of Expression of Membrane Receptors

An enzyme-linked immunosorbent assay (ELISA) (Nunc Immuno MaxiSorp, Thermo Fisher Scientific, Waltham, MA, USA) was used to quantify membrane receptor expression [68]. Samples containing cell lysates were treated with carbonate-binding buffer containing 5% non-fat milk (blocking buffer) in wells of plates, on the surface of which proteins were bound, respectively. Sample wells were then washed with phosphate-buffered saline (PBS) solution containing 0.1% Tween (PBS-T), after which samples were incubated overnight with the appropriate primary antibody (CB1 and CB2 (host: mouse) (Santa Cruz Biotechnology, Dallas, TX, USA); TRPV1 (host: mouse) (Sigma-Aldrich, St. Louis, MO, USA) and PPARγ (host: rabbit) (Invitrogen, Thermo Fisher Scientific, Waltham, MA, USA). After incubation, washing with PBS-T mixture was performed, and then the samples were treated with a mixture that blocks peroxidase activity (3% H_2_O_2_ and 3% non-fat milk in PBS). Goat anti-rabbit/mouse EnVision+ Dual Link/HRP solution (1:100) (Agilent Technologies, Santa Clara, CA, USA) was used as the secondary antibody and then incubated. After removing the secondary antibody, a chromogen solution containing 0.1 mg/mL TMB and 0.012% hydrogen peroxide was added to the samples. The reaction was stopped by the addition of 2 M sulfuric acid (H_2_SO_4_), and then absorption was measured at 450 nm, taking a reading of the value after 10 min. Membrane receptor expression was recalculated based on standard curves prepared for each compound: CB1—0.1–10 ng/mL, r^2^ −0.999; CB2—0.1–6 μg/mL, r^2^ −0.999; TRPV1-1-100 μg/mL, r^2^ −0.999; PPARγ-1-150 pg/mL, r^2^ −0.999 (TRPV1:Lifespan Biosciences, Seattle, WA, USA; PPARγ:Fine, Wuhan Test, Hubei, China; CB1:Abcam, Cambridge, UK; and CB2: Abnova, Taipei, Taiwan). The values obtained were normalized by including the protein content in the samples.

#### 2.2.12. Statistical Analysis

The obtained results were evaluated using the Shapiro–Wilk test to assess normality of distribution and presented as mean ± standard deviation (SD) for n = 5. Student’s *t*-test was used for data analysis. The performed experiments were carried out on cell lines, and non-biodiversity-reflecting technical repetitions were used as replicates. Statistical significance was defined as a *p*-value < 0.05.

## 3. Results

### 3.1. The Influence of Lipid Extracts from the Microalgae Chlorococcum amblystomatis and Nannochloropsis oceanica on Redox Balance Disturbance Caused by UVA Irradiation of Fibroblasts

The effectiveness of the action of lipid extracts from algae on the metabolism of fibroblasts was assessed in physiological conditions by introducing lipid extracts of microalgae into the medium of control fibroblasts (not exposed to external factors) and into the medium of cells after their UVA irradiation, treating the microalgae extracts as potential factors regenerating the functioning of cells at the redox balance and phospholipid metabolism levels.

**Figure 1:**
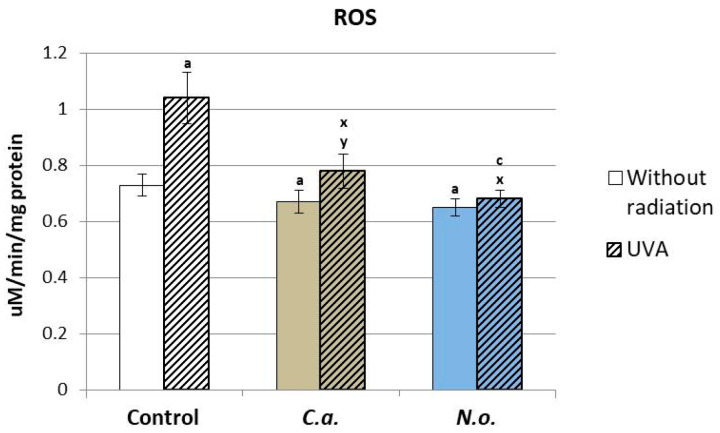
Effect of *Chlorococcum amblystomatis* (*C.a.*) or *Nannochloropsis oceanica* (*N.o.*) microalgae extract on the level of reactive oxygen species (ROS) in the following groups of fibroblasts: o control (n = 5): control cells without and after UVA irradiation [20 J/cm^2^]; o *C.a.* (n = 5): cells cultured for 24 h with *C.a.* [2 μg/mL] without irradiation and after irradiation with UVA [20 J/cm^2^]; o *N.o.* (n = 5): cells cultured for 24 h with *N.o.* [2 μg/mL] without irradiation and after irradiation with UVA [20 J/cm^2^]. Mean values ± SD and statistically significant differences for *p* < 0.05 are presented: **a**—vs. control; **c**—UVA+algae *N.o.* vs. UVA+algae *C.a.*; **x**—vs. UVA; **y**—UVA+algae *C.a.* vs. algae *C.a*.

It was found that both microalgae extracts (from *C.a.* and *N.o.*) significantly reduced the level of ROS in control cells (not exposed to UVA). They also reduced the ROS level, significantly increased by UVA radiation, to the control level. This was especially true for *N.o.* (Figure 1).

**Figure 2:**
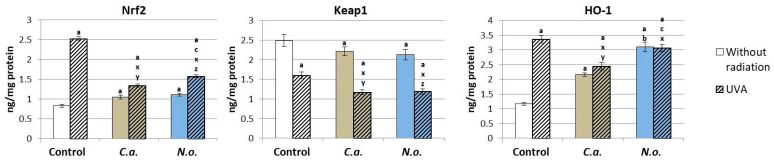
Effect of *Chlorococcum amblystomatis* (*C.a.*) or *Nannochloropsis oceanica* (*N.o.*) microalgae extract on the expression of Nrf2 transcription factor, its main product (HO-1) and inhibitor (Keap1) in the following groups of fibroblasts: o control (n = 5): control cells without and after UVA irradiation [20 J/cm^2^]; o *C.a.* (n = 5): cells cultured for 24 h with *C.a.* [2 μg/mL] without irradiation and after irradiation with UVA [20 J/cm^2^]; o *N.o.* (n = 5): cells cultured for 24 h with *N.o.* [2 μg/mL] without irradiation and after irradiation with UVA [20 J/cm^2^]. Mean values ± SD and statistically significant differences for *p* < 0.05 are presented: **a**—vs. control; **b**—algae *N.o.* vs. algae *C.a.*; **c**—UVA+algae *N.o.* vs. UVA+algae *C.a.*; **x**—vs. UVA; **y**—UVA+algae *C.a.* vs. algae *C.a.*; **z**—UVA+algae *N.o.* vs. algae *N.o*.

The introduction of lipid extracts from both types of microalgae into the control medium of fibroblasts resulted in a statistically significant increase in the level of Nrf2, which was directly related to the decrease in the level of the inhibitor of Nrf2—protein Keap-1 (Figure 2). The increase in Nrf2 levels resulted in an enhancement in the amount of HO-1 protein, which is an indicator of Nrf2 transcription efficiency. However, UVA irradiation of fibroblasts resulted in an approximately 3-fold increase in the levels of Nrf2 and HO-1 and a statistically significant decrease in the level of Keap1. The application of both types of algae (*C.a.* and *N.o.*) to the fibroblast medium after UVA irradiation significantly reduced the level of Nrf2, which, however, remained higher than in the control group, especially after the application of *N.o.* algae, which resulted in a reduction in the HO-1 level. However, the level of the Nrf2 inhibitor—Keap1—in fibroblasts exposed to microalgae (*C.a.* and *N.o.*) after exposure to UVA was further reduced compared to cells exposed to UVA.

**Figure 3:**
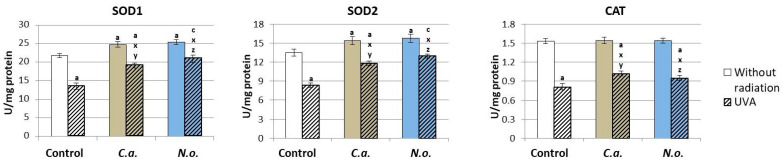
Effect of *Chlorococcum amblystomatis* (*C.a.*) or *Nannochloropsis oceanica* (*N.o.*) microalgae extracts on the activity of cytosol and mitochondrial superoxide dismutases (SOD1 and SOD2) and catalase (CAT) in the following groups of fibroblasts: o control (n = 5): control cells without and after UVA irradiation [20 J/cm^2^]; o *C.a.* (n = 5): cells cultured for 24 h with *C.a.* [2 μg/mL] without irradiation and after irradiation with UVA [20 J/cm^2^]; o *N.o.* (n = 5): cells cultured for 24 h with *N.o.* [2 μg/mL] without irradiation and after irradiation with UVA [20 J/cm^2^]. Mean values ± SD and statistically significant differences for *p* < 0.05 are presented: **a**—vs. control; **c**—UVA+algae *N.o.* vs. UVA+algae *C.a.*; **x**—vs. UVA; **y**—UVA+algae *C.a.* vs. algae *C.a.*; **z**—UVA+algae *N.o.* vs. algae *N.o*.

Lipid extracts from microalgae *C.a.* and *N.o.* introduced into the cell medium contributed to a significant increase in the activity of superoxide dismutase—a basic antioxidant enzyme (both cytosolic and mitochondrial isoforms—SOD1/2) in control fibroblasts (Figure 3). Above all, however, they caused a significant increase in the reduced activity of all three assessed antioxidant enzymes (SOD1/2 and CAT) in fibroblasts exposed to UVA radiation.

**Figure 4:**
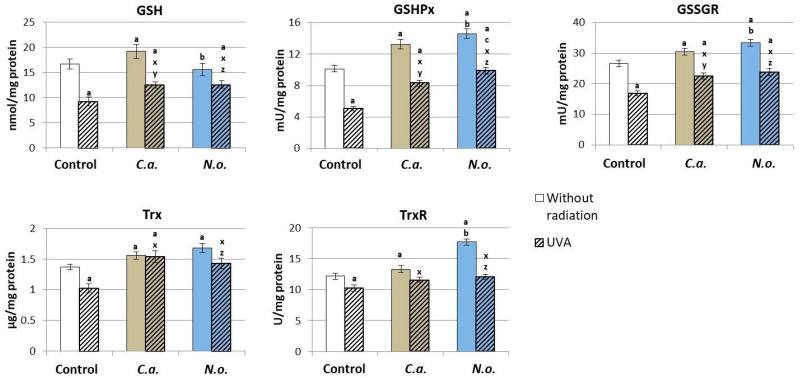
Effect of *Chlorococcum amblystomatis* (*C.a.*) or *Nannochloropsis oceanica* (*N.o.*) microalgae extracts on the elements of glutathione and thioredoxin systems in the following groups of fibroblasts: o control (n = 5): control cells without and after UVA irradiation [20 J/cm^2^]; o *C.a.* (n = 5): cells cultured for 24 h with *C.a.* [2 μg/mL] without irradiation and after irradiation with UVA [20 J/cm^2^]; o *N.o.* (n = 5): cells cultured for 24 h with *N.o.* [2 μg/mL] without irradiation and after irradiation with UVA [20 J/cm^2^]. Mean values ± SD and statistically significant differences for *p* < 0.05 are presented: **a**—vs. control; **b**—algae *N.o.* vs. algae *C.a.*; **c**—UVA+algae *N.o.* vs. UVA+algae *C.a*.; **x**—vs. UVA; **y**—UVA+algae *C.a.* vs. algae *C.a.*; **z**—UVA+algae *N.o.* vs. algae *N.o*.

Lipid extracts from the microalgae *C.a.* contributed to the increase in the level/activity of all ingredients of the glutathione system and Trx from the thioredoxin system in the control fibroblasts (Figure 4). However, the application of the *N.o.* extract caused an increase in GSHPx, GSSGR and TrxR activity. The application of the above-mentioned microalgae extracts to the fibroblast medium after UVA irradiation caused an increase in the level of all analyzed parameters of the glutathione and thioredoxin systems compared to reduced values in UVA-irradiated keratinocytes.

### 3.2. The Influence of Lipid Extracts from the Microalgae Chloroccocum amblystomatis and Nannochloropsis oceanica on Lipid Peroxidation Caused by UVA Irradiation of Fibroblasts

**Figure 5:**
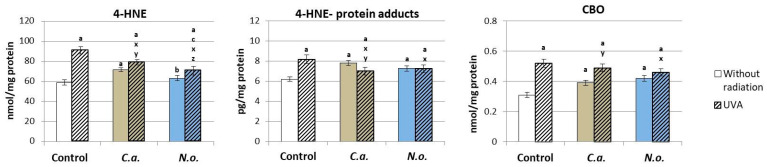
Effect of *Chlorococcum amblystomatis* (*C.a.*) or *Nannochloropsis oceanica* (*N.o.*) microalgae extracts on the level of 4-HNE, 4-HNE-protein adducts and protein carboxyl groups (CBO) in the following fibroblast groups: o control (n = 5): control cells without and after UVA irradiation [20 J/cm^2^]; o *C.a.* (n = 5): cells cultured for 24 h with *C.a.* [2 μg/mL] without irradiation and after irradiation with UVA [20 J/cm^2^]; o *N.o.* (n = 5): cells cultured for 24 h with *N.o.* [2 μg/mL] without irradiation and after irradiation with UVA [20 J/cm^2^]. Mean values ± SD and statistically significant differences for *p* < 0.05 are presented: **a**—vs. control; **b**—algae *N.o.* vs. algae *C.a.*; **c**—UVA+algae *N.o.* vs. UVA+algae *C.a.*; **x**—vs. UVA; **y**—UVA+algae *C.a.* vs. algae *C.a.*; **z**—UVA+algae *N.o.* vs. algae *N.o*.

The consequence of changes in the redox system after the use of microalgae lipid extracts was changes in the process of lipid peroxidation and protein modification. It was found that the microalgae *C.a.* significantly increased the level of 4-HNE, 4-HNE-protein adducts and carbonyl groups in proteins that were assessed (Figure 5), while the lipid extract of microalgae *N.o.* increased only the level of 4-HNE-protein adducts and protein carbonyl groups. UVA irradiation of fibroblasts resulted in a significant increase in the level of all analyzed parameters. However, the use of C.a. extract after the irradiation of cells caused a significant reduction in the level of 4-HNE and 4-HNE-protein adducts, while the *N.o.* extract contributed to a reduction in the level of all analyzed parameters. However, reducing the levels of the analyzed parameters did not result in achieving the values in the control group.

**Figure 6:**
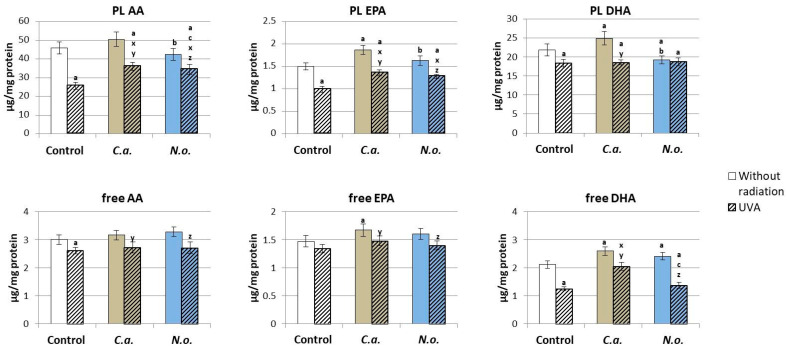
Effect of *Chlorococcum amblystomatis* (*C.a.*) or *Nannochloropsis oceanica* (*N.o.*) microalgae extracts on the level of free and phospholipid (PL) non-saturated fatty acids in the following groups of fibroblasts: o control (n = 5): control cells without and after UVA irradiation [20 J/cm^2^]; o *C.a.* (n = 5): cells cultured for 24 h with *C.a.* [2 μg/mL] without irradiation and after irradiation with UVA [20 J/cm^2^]; o *N.o.* (n = 5): cells cultured for 24 h with *N.o.* [2 μg/mL] without irradiation and after irradiation with UVA [20 J/cm^2^]. Mean values ± SD and statistically significant differences for *p* < 0.05 are presented: **a**—vs. control; **b**—algae *N.o.* vs. algae *C.a.*; **c**—UVA+algae *N.o.* vs. UVA+algae *C.a.*; **x**—vs. UVA; **y**—UVA+algae *C.a.* vs. algae *C.a.*; **z**—UVA+algae *N.o.* vs. algae *N.o*.

The treatment of the control fibroblasts with lipid extracts from microalgae *C.a.* caused an increase in the level of free and phospholipid fatty acids (EPA and DHA) (Figure 6). However, the use of *N.o.* caused an increase in the level of free and a decrease in the level of phospholipid DHA. The consequence of UVA irradiation was a significant reduction in the level of phospholipid PUFAs (AA, EPA and DHA) and free PUFAs (AA, DHA). However, the application of lipid extracts from microalgae *C.a.* or *N.o.* to the medium of the above-mentioned fibroblasts promoted an increase in the level of phospholipid AA and EPA as well as free DHA after the use of *N.o.*

**Figure 7:**
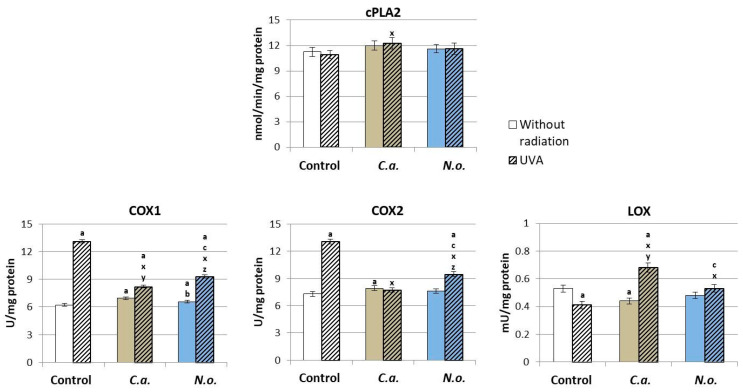
Effect of *Chlorococcum amblystomatis* (*C.a.*) or *Nannochloropsis oceanica* (*N.o.*) microalgae extracts on the activity of cyclooxygenase-1/2, cytosolic phospholipase A2 and 5-lipoxygenase (5-LOX) in the following fibroblast groups: o control (n = 5): control cells without and after UVA irradiation [20 J/cm^2^]; o *C.a.* (n = 5): cells cultured for 24 h with *C.a.* [2 μg/mL] without irradiation and after irradiation with UVA [20 J/cm^2^]; o *N.o.* (n = 5): cells cultured for 24 h with *N.o.* [2 μg/mL] without irradiation and after irradiation with UVA [20 J/cm^2^]. Mean values ± SD and statistically significant differences for *p* < 0.05 are presented: **a**—vs. control; **b**—algae *N.o.* vs. algae *C.a.*; **c**—UVA+algae *N.o.* vs. UVA+algae *C.a.*; **x**—vs. UVA; **y**—UVA+algae *C.a.* vs. algae *C.a.*; **z**—UVA+algae *N.o.* vs. algae *N.o*.

Regardless of the effect of microalgae lipid extracts on ROS-dependent phospholipid metabolism, the enzyme-dependent metabolism of these compounds was also changed. However, neither the use of microalgae nor UVA irradiation caused significant changes in the activity of the phospholipase A2 enzyme responsible for the release of free fatty acids from membrane phospholipids, but the introduction of lipid extract from microalgae *C.a.* into the fibroblast medium caused a significant increase in COX1 and COX2 activity and a decrease in 5-LOX activity (Figure 7), while the microalgae extract *N.o.* increased only the activity of COX1. The consequence of UVA radiation was an increase in the activity of cyclooxygenase isoenzymes (COX1 and COX2), but not 5-lipoxygenase (5-LOX) (Figure 7). The application of *C.a.* microalgae extract after UVA irradiation increased the activity of COX1 and 5-LOX, while *N.o.* microalgae extract decreased COX1/2 and increased LOX-5 activity compared to UVA-irradiated fibroblasts, but only 5-LOX activity reached the control level.

**Figure 8:**
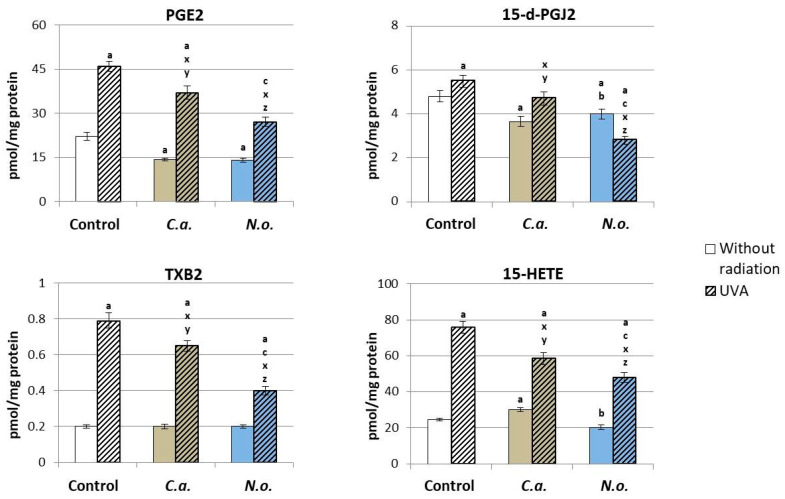
Effect of *Chlorococcum amblystomatis* (*C.a.*) or *Nannochloropsis oceanica* (*N.o.*) microalgae extracts on the level of eicosanoids in the following fibroblast groups: o control (n = 5): control cells without and after UVA irradiation [20 J/cm^2^]; o *C.a.* (n = 5): cells cultured for 24 h with *C.a.* [2 μg/mL] without irradiation and after irradiation with UVA [20 J/cm^2^]; o *N.o.* (n = 5): cells cultured for 24 h with *N.o.* [2 μg/mL] without irradiation and after irradiation with UVA [20 J/cm^2^]. Mean values ± SD and statistically significant differences for *p* < 0.05 are presented: **a**—vs. control; **b**—algae *N.o.* vs. algae *C.a.*; **c**—UVA+algae *N.o.* vs. UVA+algae *C.a.*; **x**—vs. UVA; **y**—UVA+algae *C.a.* vs. algae *C.a.*; **z**—UVA+algae *N.o.* vs. algae *N.o*.

Changes in the activity of lipolytic enzymes after the use of lipid extracts from microalgae or the irradiation of cells with UVA promoted changes in the level of lipid mediators that are products of these enzymes’ actions. The application of lipid extract from microalgae *C.a.* to the medium of control fibroblasts resulted in a significant reduction in the level of PGE2, 15-d-PGJ2 and an increase in the level of 15-HETE, while the use of lipid extract from microalgae *N.o.* contributed to reducing the levels of all above-mentioned eicosanoids (Figure 8). UVA radiation, by increasing the activity of enzymes responsible for the metabolism of PUFAs, contributed to an increase in the level of products of the metabolism of these acids, including the products of COXs (PGE2, 15-d-PGJ2 and TXB2) and LOX (15-HETE). Both types of microalgae reduced the level of all assessed eicosanoids increased by UVA exposure, with the level of 15-d-PGJ2 being reduced below the value in the control group.

**Figure 9:**
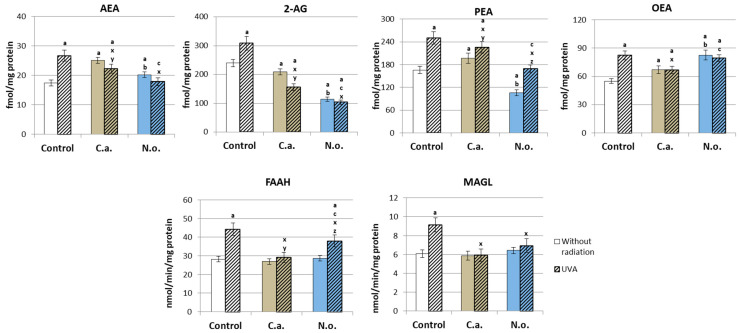
Effect of *Chlorococcum amblystomatis* (*C.a.*) or *Nannochloropsis oceanica* (*N.o.*) microalgae extracts on the level of endocannabinoids (anandamide (AEA), 2-arachidonylglycerol (2-AG) and derivative compounds—palmitoylethanolamide (PEA) and oleoylethanolamide (OEA)) and the activity of AEA and 2-AG metabolizing enzymes (FAAH and MAGL) in the following fibroblast groups: o control (n = 5): control cells without and after UVA irradiation [20 J/cm^2^]; o *C.a.* (n = 5): cells cultured for 24 h with *C.a.* [2 μg/mL] without irradiation and after irradiation with UVA [20 J/cm^2^]; o *N.o.* (n = 5): cells cultured for 24 h with *N.o.* [2 μg/mL] without irradiation and after irradiation with UVA [20 J/cm^2^]. Mean values ± SD and statistically significant differences for *p* < 0.05 are presented: **a**—vs. control; **b**—algae *N.o.* vs. algae *C.a.*; **c**—UVA+algae *N.o.* vs. UVA+algae *C.a.*; **x**—vs. UVA; **y**—UVA+algae *C.a.* vs. algae *C.a.*; **z**—UVA+algae *N.o.* vs. algae *N.o*.

As a result of the action of enzymes from the phospholipase group, phospholipid PUFAs were metabolized into endocannabinoids, including the recommended ones: AEA, 2-AG, PEA and OEA. However, the use of lipid extract from *C.a.* caused an increase in the levels of AEA, PEA and OEA, and the *N.o.* extract increased the levels of AEA and OEA and decreased the levels of 2AG and PEA (Figure 9). UVA radiation significantly increased the level of all analyzed endocannabinoids. However, post-UVA application of the microalgae extract resulted in a significant reduction in the elevated levels of all endocannabinoids analyzed when the microalgae *N.o.* promoted the return of AEA and PEA levels to those in the control group, and 2-AG and OEA levels remained the same as in the group treated with only *N.o.* microalgae.

**Figure 10:**
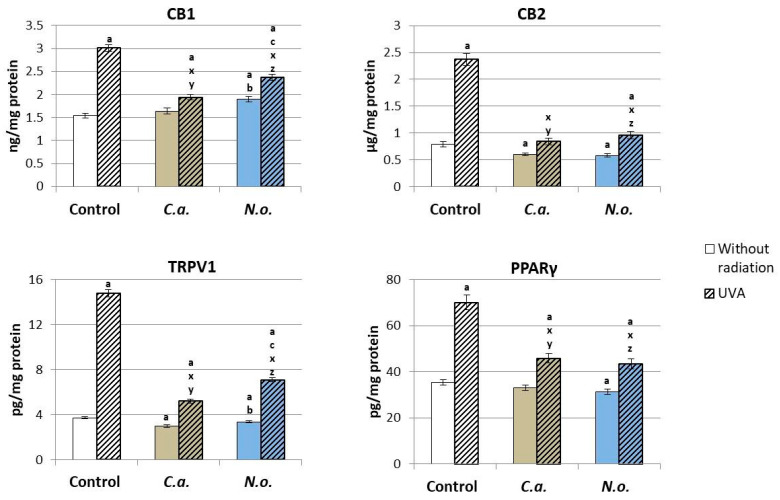
Effect of *Chlorococcum amblystomatis* (*C.a.*) or *Nannochloropsis oceanica* (*N.o.*) microalgae extracts on the expression of G-protein-coupled receptors (CB1, CB2, PPARγ, TRPV1) in the following fibroblast groups: o control (n = 5): control cells without and after UVA irradiation [20 J/cm^2^]; o o *C.a.* (n = 5): cells cultured for 24 h with *C.a.* [2 μg/mL] without irradiation and after irradiation with UVA [20 J/cm^2^]; o *N.o.* (n = 5): cells cultured for 24 h with *N.o.* [2 μg/mL] without irradiation and after irradiation with UVA [20 J/cm^2^]. Mean values ± SD and statistically significant differences for *p* < 0.05 are presented: **a**—vs. control; **b**—algae *N.o.* vs. algae *C.a.*; **c**—UVA+algae *N.o.* vs. UVA+algae *C.a.*; **x**—vs. UVA; **y**—UVA+algae *C.a.* vs. algae *C.a.*; **z**—UVA+algae *N.o.* vs. algae *N.o*.

Changes in the level of lipid mediators from the group of endocannabinoids and eicosanoids modified the expression of membrane G-protein-coupled receptors. Consequently, the use of the lipid extract of *N.o.* microalgae promoted an increase in the expression of the CB1 receptor (Figure 10). The use of extracts from both types of microalgae reduced the expression of CB2, TRPV1 and PPARγ receptors. However, the exposure of fibroblasts to UVA radiation contributed to an increase in the expression of all analyzed membrane receptors, and the use of extracts from both types of microalgae after UVA irradiation of cells resulted in a significant reduction in the level of all determined receptors, but their level remained higher than in the control group.

**Figure 11:**
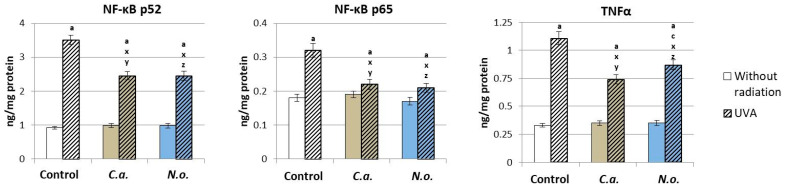
Effect of *Chlorococcum amblystomatis* (*C.a.*) or *Nannochloropsis oceanica* (*N.o.*) microalgae extracts on the level of subunits of transcription factor NF-κB (p52 and p65) and cytokine TNFα in the following fibroblast groups: o control (n = 5): control cells without and after UVA irradiation [20 J/cm^2^]; o o *C.a.* (n = 5): cells cultured for 24 h with *C.a.* [2 μg/mL] without irradiation and after irradiation with UVA [20 J/cm^2^]; o *N.o.* (n = 5): cells cultured for 24 h with *N.o.* [2 μg/mL] without irradiation and after irradiation with UVA [20 J/cm^2^]. Mean values ± SD and statistically significant differences for *p* < 0.05 are presented: **a**—vs. control; **c**—UVA+algae *N.o.* vs. UVA+algae *C.a.*; **x**—vs. UVA; **y**—UVA+algae *C.a.* vs. algae *C.a.*; **z**—UVA+algae *N.o.* vs. algae *N.o*.

The observed changes in the level and effectiveness of Nrf2 and the expression of G-protein-coupled receptors under the influence of physical (UVA) and chemical (algae) factors, presented in the previous figures, contributed to the modification expression/efficiency of inflammatory signaling of the NF-κB transcription factor (Figure 11). The use of *C.a.* and *N.o.* microalgae extracts did not significantly affect the levels of NF-κB and cytokine TNFα in control fibroblasts. However, UVA radiation significantly increased the expression of both NF-κB subunits (p52 and p65) and the product of NF-κB transcriptional activity, TNFα, while microalgae extracts decreased the levels of both NF-κB subunits and TNFα, but the levels of the assessed parameters remained higher than in the control group.

## 4. Discussion

Skin cells, which constitute a barrier between the human body and the surrounding external environment, are subject to constant physiological metabolic changes but also to metabolic modifications as a result of the action of exogenous physicochemical factors, including solar radiation. The metabolic response of cells usually begins with modifications of redox balance and pro-inflammatory signaling [1]. The consequence of this is the search for substances/compounds, especially of natural origin, that could ensure metabolic stability at the level of skin cells. The group of natural substances containing antioxidant and anti-inflammatory components includes marine and freshwater algae, which are intensively researched as potential natural regulators of cellular metabolism and are also of interest to the pharmaceutical and nutraceutical industries [69]. Due to the high content of PUFAs and other biologically active compounds, including those with antioxidant and anti-inflammatory properties regulating cellular metabolism, marine microalgae *Nannochloropsis oceanica* and freshwater microalgae *Chlorococcum amblystomatis* are of scientific interest [70,71,72,73,74].

### 4.1. Protective Effect of Marine and Freshwater Microalgae on Skin Fibroblasts

The results of this study show that, under control conditions, lipid extracts from both microalgae used in this study (marine and freshwater) exhibit antioxidant activity in the human skin fibroblast environment, with the lipid extract from the marine microalgae *Nannochloropsis oceanica* appearing to be more effective. Literature data confirm that extracts of both microalgae in vitro show significant antioxidant activity assessed by the efficiency of scavenging the 2,20-azinobis-3-ethylbenzothiazoline-6-sulfonic acid radical (ABTS•+) and the α,α-diphenyl-β-radical picrylhydrazyl (DPPH•) [26,74]. In the present study, lipid extracts of these microalgae induced a reduction in the level of reactive oxygen species (ROS) in fibroblasts, which, in the context of the above literature data, may be a consequence of both direct scavenging of ROS as well as increased effectiveness of cellular antioxidants. The lipid extracts of microalgae regulate the level and effectiveness of the nuclear transcription factor Nrf2, which is responsible for the transcription and biosynthesis of antioxidant proteins. Under physiological conditions, Nrf2 interacts with the Kelch-like ECH-associated protein-1 (Keap1), which, acting as a cytoplasmic inhibitor of Nrf2, directs the transcription factor to ubiquitination and proteasomal degradation [75]. The use of *C.a.* and *N.o.* lipid extracts significantly reduces the level of Keap1 protein, which promotes an increase in transcriptional efficiency of Nrf2 assessed by the level of heme oxygenase (HO-1), a basic indicator of Nrf2 activity, and an enzyme, which both itself and through degradation products reveals antioxidant effects [75]. At the same time, it is observed that the *N.o.* microalgae extract is much more effective than the *C.a.* extract at the stage of transcription of antioxidant proteins, and, consequently, more strongly increases the antioxidant efficiency of fibroblasts. This is visible at the level of biological activity of other classic cellular antioxidants, including the basic antioxidant enzyme, superoxide dismutase, which is responsible for the metabolism of superoxide anion radicals both in the cytosol (SOD1) and in the mitochondrial matrix (SOD2) [76].

At the same time, microalgae extracts also increase both enzymatic activity and the level of non-enzymatic elements of two main cytosolic antioxidant systems: thioredoxin- and glutathione-dependent systems. Because both of these systems participate in maintaining the stability of membrane phospholipids, the intensification of their antioxidant activities promotes the effective protection of membrane phospholipids, especially at the level of unsaturated fatty acids, but also at cellular proteins against potential oxidation [77]. Literature data showed the same direction of action of the lipid extract from marine algae *N.o.* on human skin keratinocytes [41]. This direction of action is probably the result of the action of lipo- and hydrophilic vitamins contained in microalgae (including α-tocopherol and ascorbic acid) as well as cooperating with each other, as well as pigments from the group of chlorophylls and carotenoids (including astaxanthin, violaxanthin and zeaxanthin), which also reveal significant antioxidant effects [78,79,80]. Water-soluble vitamin C, due to the double bond in the ring and four hydrophilic groups, has an antioxidant effect in hydrophilic environments, acting as a cofactor of enzymatic reactions as well as supporting the regeneration of vitamin E, which is involved in the protection of phospholipids [81]. Moreover, this vitamin can also capture radical forms, and its lipophilic nature favors the protection of the environment of membrane phospholipids [82]. Literature data also indicate that lipophilic astaxanthin, which is present mainly in freshwater algae *C.a.* [27], easily penetrates cells and is one of the strongest antioxidants, the effectiveness of which is estimated to be up to 100 times higher than that of α-tocopherol [83]. Moreover, it was found that astaxanthin, among others, participates in the activation and effectiveness of the Nrf2 transcription factor [84,85], which corresponds to these results. The antioxidant and anti-inflammatory effects are also supported by β-carotene and chlorophyll α and β present in the tested microalgae [30,86].

Strengthening the endogenous antioxidant systems of fibroblasts by antioxidants contained in microalgae promotes the protection of membrane phospholipids. The lack of changes in the activity of phospholipase A2 after the use of microalgae favors the stabilization of the phospholipid structure of fibroblast membranes, despite the observed increase in the level of free EPA and DHA under the influence of both types of microalgae. This may be favored by the high content of PUFAs in the lipid extracts of microalgae, and according to literature data, *N.o.* microalgae contain PUFAs, such as EPA and AA [24], which probably enrich the phospholipid structure of fibroblasts. However, *C.a.* has a stronger effect, significantly increasing the levels of both phospholipids and free EPA and DHA [26,27,30]. It can also be suggested that the lipophilic microenvironment created in the culture medium by used microalgae may promote the biosynthesis of both free and phospholipid PUFAs in fibroblasts. However, this situation also favors the observed increased lipid peroxidation, with an increase in the level of the basic lipid peroxidation product (4-HNE) and, consequently, an increase in the level of its adducts with proteins. However, the observed changes in the level of fatty acids are also related to the activity of enzymes involved in the metabolism of PUFAs to lipid mediators, mainly COX1/2. The microalgae *C.a.*, by increasing COX activity, especially its constitutive isoform, promotes the growth of lipid mediators, including eicosanoids. However, this is not entirely clear, because the level of free PUFAs also depends on enzymes that metabolize endocannabinoids (FAAH and MAGL) [87], whose activity is not dependent on the presence of microalgae. Endocannabinoid levels after microalgae use show a mixed trend, including an increase (AEA and OEA), but also a decrease, in 2-AG levels and an increase in PEA levels after *C.a.* and a decrease in the level of this endocannabinoid after the use of *N.o.* The consequence of such a metabolic response on the part of fibroblasts may be additional modifications in the level of free PUFAs.

However, the obtained results regarding the enzymatic activity of FAAH and MAGL do not explain the increase in the content of the free fatty acids AA, EPA and DHA after the use of an extract from *C.a.* lower in these fatty acids. This fact may, at least partially, be explained by the observed tendency to reduce the level of most of the eicosanoids determined (PGE2, 15-d-PGJ2 and TXB2), with the exception of 15-HETE, the level of which increased after the use of microalgae, which may confirm the above reasoning. At the same time, it should be taken into account that 15-HETE is a product of the action of LOXs, among which the enzyme assessed in these studies had reduced activity under the influence of the tested *C.a.* microalgae extract, while a reduced level of 15-HETE was observed in the case of the *N.o.* extract resulting from reduced LOX activity. However, it should be taken into account that 15-HETE is also generated by the action of other isoenzymes from the lipoxygenases group. In previous studies on keratinocytes exposed to the lipid extract of *N.o.*, a similar direction of change in the levels of both LOX-5 and 15-HETE was observed [88]. Inhibition of LOX activity was also observed in the case of in vivo activation of allergen-induced leukocytes, in which there was a significant release of 15-HETE, because it is believed that a high concentration of this eicosanoid may also be responsible for the reduction in LOX-5 activity [89], which partially may explain the observations of this study. Additionally, one can mention data obtained in studies conducted on mouse colon tumors, where it was observed that in LOX-12/15 knockout animals, there was no reduction in the level of 15-HETE in isolated macrophages compared to cells derived from wild-type mice [90]. The above literature reports and observations from the present research may suggest two hypotheses, namely that 15-HETE is produced via an alternative metabolic pathway independent of LOX and that the increased generation of 15-HETE from arachidonic acid occurs at the expense of the production of other metabolites (e.g., 12-HETE); however, both suggestions require confirmation. The observed changes in phospholipid metabolism (both enzymatic and ROS-dependent) are interesting because both 15-HETE and 4-hydroxynonenal (4-HNE) are produced as a result of the transformation of the same precursor—15-HPETE [59]. The effect of glutathione peroxidase on 15-HPETE is the formation of the above-mentioned anti-inflammatory eicosanoid. However, as a result of the action of free radicals, 15-HPETE is transformed into 4-HNE, which also acts as an important transmitter of signaling processes in the cell, including by activating inflammasomes [91]. Under physiological conditions, 4-HNE is removed from cells through conjugation with glutathione, taking advantage of the high reactivity of 4-HNE related to the presence of three reactive groups (double bond, hydroxyl group and carbonyl group) and the resulting rapid binding to amino acids such as cysteine (found, among others, in the GSH molecule), as well as histidine, lysine and arginine [92]. Therefore, from a cellular point of view, it is important to monitor the concentration of this aldehyde together with the level of adducts it forms with peptides and proteins. These results indicate that both *Chlorococcum amblystomatis* and *Nannochloropsis oceanica* extracts lead to an increased generation of 4-HNE and its adducts with proteins; however, in the case of both parameters, these levels do not indicate obvious pathology. It should also be highlighted that 4-HNE, by binding to the Cys^151^ and Cys^288^ of Keap1, promotes increased translocation of Nrf2 to the cell nucleus and its increased efficiency [93], as suggest the present results. Taking into account the fact that the tested extracts increase the level of protein carbonyl groups (CBO), it can be suggested that the lipid extracts used may lead to disturbances in protein metabolism, which, however, requires further analyses in the field of proteomics.

The metabolism of phospholipid unsaturated fatty acids via N-acyltransferases (NAT) and the appropriate C- and D-phospholipases leads to the generation of 2-arachidonoglycerol (2-AG) and N-arachidonoethanolamine (AEA), which are, respectively, ligands for cannabinoid receptors 1/2 (CB1/2). The interaction between the emerging lipid mediators, including endocannabinoids, but also eicosanoids and G-protein-coupled receptors, is extremely important from the point of view of cell homeostasis, because they modulate, among other processes, redox balance and the development of inflammation [16,94]. Reducing the level of pro-inflammatory 2-AG with a simultaneous increase in the concentration of AEA and its derivatives (PEA and OEA) as a result of the action of the tested microalgae extracts should lead to a reduction in the cellular inflammatory response. However, CB1/2 receptor levels indicate that both *C.a*. and *N.o*. increase the expression of the pro-inflammatory CB1 receptor while decreasing the expression of CB2, TRPV1 and PPARγ receptors. Therefore, it is possible that the regulation of redox balance and inflammatory signaling fibroblasts by both microalgae species under physiological conditions may be at least partially related to the endocannabinoid system, which, however, requires further analysis.

### 4.2. Regenerative Effect of the Microalgae Nannochloropsis oceanica and Chlorococcum amblystomatis on the Metabolism of UVA-Irradiated Fibroblasts

Compounds that have a regulatory effect on the redox balance and pro-inflammatory signaling contained in marine microalgae and freshwater not only support the functioning of fibroblasts in physiological conditions, but, as shown by this study, also have a regenerative effect on cells previously exposed to the action of an environmental physical factor such as UVA radiation, which due to its energetic properties reaches up to the dermis, disturbing the metabolism of its cells, including fibroblasts [95]. At the same time, however, UVA radiation, due to its ability to inhibit the cell cycle, is used in phototherapy of skin diseases such as psoriasis [96]. However, regardless of its origin, UVA radiation typically activates a pro-oxidant and pro-inflammatory response, which promotes the modification of the structure and function of macromolecules (proteins, lipids and nucleic acids) throughout the skin, including fibroblasts [97]. However, due to the vascularization of the dermis, the metabolic effects of UVA radiation may also be transferred to the systemic level [19,21]. Since disorders of phospholipid metabolism resulting from the action of UVA radiation on skin cells lead to increased production of numerous lipid mediators from the endocannabinoids and eicosanoids group, this may favor metabolic disorders resulting from an increased pro-oxidant and pro-inflammatory state. Therefore, we are constantly looking for compounds/substances that would limit the negative effects of this radiation and at the same time would not negatively affect healthy cells, as is the case with algae extracts.

The results of the conducted research indicate that the introduction of lipid extracts from microalgae into the culture medium of fibroblasts previously exposed to UVA rays affects the regeneration of cellular metabolism. This is seen in the regulation of the redox balance of these cells. It has been shown that lipid extracts from marine microalgae reduce, similarly to control fibroblasts, only more effectively, the production of ROS caused by UVA and increase the activity of antioxidant enzymes in fibroblasts. In the case of marine microalgae *N.o.*, even the restoration of the physiological values of the ROS level was observed, which may suggest a directionally more effective antioxidant effect of these algae compared to freshwater microalgae *C.a.* This situation may be related to the antioxidant effect of xanthophylls, the amount of which in microalgae *N.o.* is up to 10 times higher compared to microalgae *C.a.* [27,86]. For previous studies, using violaxanthin obtained from the microalgae *Eustigmatos* cf. *polyphem* showed significant antioxidant activity of this carotenoid, manifested by the effectiveness of scavenging ABTS·+ and DPPH· radicals [98]. It was also shown that despite significant structural similarity, violaxanthin is a more effective free radical scavenger than lutein and β-carotene [99], which occur in larger amounts in microalgae *C.a.* [27]. The effect of the antioxidant effect of the extracts of the tested microalgae is also an increase in the concentration of non-enzymatic elements of the cell’s antioxidant response, such as thioredoxin and glutathione, which, due to their protective effect on phospholipids, translates into a significant reduction in the formation of oxidative modifications of lipids compared to cells exposed only to UVA radiation. It should be emphasized that previous studies have already demonstrated the ability to limit the production and effect of ROS induced by UVB radiation in fibroblasts by the microalgae *Ettlia* sp. *YC001* [100] and the regenerative effect (including antioxidant) of the lipid extract from *Nannochloropsis oceanica* in relation to keratinocytes exposed to UVB radiation [41].

The results obtained in this study indicate that the tested microalgae extracts regenerate the redox balance of fibroblasts not only directly by reducing the level of ROS and, consequently, preventing oxidative modifications of the structure of antioxidants, but also by significantly regulating the physiological expression of transcription factors, including Nrf2, which is involved in the biosynthesis of antioxidant proteins, and NF-κB, which regulates pro-inflammatory signaling, the level of which significantly increases under the influence of UVA [101,102]. Regardless of the direct effect on the level of Nrf2 expression, a decrease in the level of the cytosolic Nrf2 inhibitor—protein Keap1—is observed, which overall promotes an increase in the level of HO-1, but to a significantly lesser extent than after UVA. The same direction of changes also applies to TNFα, although both types of algae significantly reduce the level of this cytokine compared to fibroblasts irradiated with UVA. Therefore, the increased transcriptional efficiency of Nrf2 promotes the reduction in the action of the NF-κB transcription factor, as shown by previous studies on keratinocytes [41]. It is suggested that such an effect may be related to the presence of the above-mentioned astaxanthin and other xanthophylls in the tested microalgae, which, like chlorophyll, mainly function by blocking the action of the cytosolic inhibitor, Keap1, promoting the biological effectiveness of the Nrf2 transcription factor and, consequently, promoting an increase in the activity of classic antioxidant enzymes (e.g., CAT, SODs, GSHPx, TrxR), while simultaneously increasing the level of factors related to cell apoptosis, including caspase 3 and 9 and Bax protein, and reducing the level of Bcl2 [103,104]. Since disruption of redox balance by UVA radiation promotes apoptosis, the tendency to reduce ROS levels and recover antioxidant levels/activities indicates a tendency for redox balance to return after microalgae treatment. In this context, the increase in the enzymatic activity of TrxR in fibroblasts regenerated using both types of microalgae after UVA irradiation deserves special attention, as both the reduction in the level of ROS—which intensifies apoptosis—and the increase in the level of Trx (which, by binding apoptosis signal-regulating kinase (ASK-1) significantly reduces p38 protein secretion [105]) regulate the level of pro-apoptotic Bax protein, thus preventing excessive apoptosis [77]. At the same time, the effectiveness of the thioredoxin system is of particular importance in the detoxification of harmful metabolites such as lipid peroxides, as well as in the regulation of gene expression and modulation of cellular signaling pathways [106,107]. An equally important function of this system is the regulation of the expression of many proteins, including apoptosis-regulating kinase (ASK-1) [77,108], and since it has been shown that the Trx-dependent system may contribute to both the intensification of angiogenesis and cell apoptosis, they have important regenerative role compounds that prevent excessive activation of the Trx-dependent system, including polyphenol compounds that inactivate elements of the thioredoxin system [109,110], which are also components of algae.

Since the effect of UVA radiation on fibroblasts leads to a disturbance of the redox balance with a shift towards oxidative conditions, it favors ROS interactions, especially with polyunsaturated fatty acids [14], as is confirmed by these results, which show a reduction in the level of phospholipid PUFAs (AA, DHA and EPA) and free PUFAs (AA and DHA). Moreover, UV-induced oxidative stress accelerates lipid extract component inflow into cells, based on both passive-diffusion as well as protein-mediated mechanisms, involving membrane-associated fatty acid-binding proteins [111] and lipid transfer proteins (LTPs) [112]. Taking into account the structural and functional similarity of phospholipid components of lipid extracts from the microalgae, this confirms their regenerative effect with respect to phospholipid PUFAs, including AA and EPA, after the use of both microalgae and free DHA after the use of *C.a.* The increased content of arachidonic and eicosapentaenoic acids after the use of *N.o.* extract may be a direct consequence of their significant content in these microalgaes (almost 10% and 30% for AA and EPA, respectively) [35]. However, the increase in the free DHA level may be related to the increase in phospholipase A2 activity by the *C.a.* extract, but this statement requires further verification. Therefore, it is highly probable that changes in the level of PUFAs under the influence of UVA are the result of increased metabolic reactions, including ROS-dependent and enzymatic ones. The effect of ROS-dependent reactions is the oxidative fragmentation of PUFA chains, generating α,β-unsaturated, reactive aldehydes and oxidative cyclization products of PUFAs [18]. The effectiveness of antioxidant components of microalgae, especially *N.o.*, promotes a reduction in both the level of 4-HNE and its adducts with proteins, as well as reduces direct oxidative modifications of proteins assessed on the basis of the level of carbonyl groups. This may be due to the presence and antioxidant activity of lipophilic antioxidants as well as vitamin C found in the microalgae *N.o.* [41,113]. Previous data have shown that treatment of fibroblasts with ascorbic acid under conditions of UV-induced oxidative stress reduces the formation of lipid peroxidation products and their further interactions, including reducing the level of carbonyl groups and the amount of another oxidative product of proteins—dityrosine [46].

Oxidative conditions observed in fibroblasts exposed to UVA also favor, through changes in the activity of enzymes responsible for the metabolism of free PUFAs, modification of the enzymatic metabolism of these acids. The effect of UVA radiation promotes an increase in the level of lipid mediators from the endocannabinoids and eicosanoids group, and the use of *N.o.* and *C.a.* reduces the level of all tested lipid mediators and the expression of all tested receptors, the level of which increases significantly after UVA irradiation. Microalgae *C.a.* reduce COX1/2 activity, and at the same time they significantly increase LOX-5 activity. This response may be related to the higher content of linoleic acid (C18:2(n-6), LA) which occurs in the microalgae *C.a.* [27,35]. As a result of the oxidation of this acid, linoleic acid hydroperoxide 13-HPODE is formed, which, as a lipid mediator, has the ability to activate LOXs [114]. Moreover, EPA, constituting approximately three percent of the biomass of the microalgae *N.o.* [115], may compete with AA as a substrate in the enzymatic metabolism catalyzed by COXs and LOXs, which may consequently reduce the generation of AA metabolites in fibroblasts [116]. EPA, in smaller amounts, is also present in extracts of the microalgae *C.a.*, which inhibit COX-2 activity [24,25]. Additionally, extracts of both microalgae contain lutein, which reduces lipid peroxidation (assessed as MDA), as well as levels of eicosanoids (PGE2, LTB4 and LTC4) and C-reactive protein and cytokines (TNF-α, IL1-β and MCP-1), and also increases the activity of antioxidant enzymes in the blood serum of rats with cataracts [117]. It was found that these effects were further enhanced by the simultaneous use of EPA and DHA [118], which corresponds to the situation with the use of microalgae extracts in the present study.

The introduction of lipid extracts of both types of microalgae (marine and freshwater) into the fibroblast environment also reduces the level of representatives of another group of lipid mediators, endocannabinoids, with the *Nannochloropsis oceanica* extract significantly reducing the level of 2-AG. This effect may be attributed to the presence of EPA in this extract, which can potentially inhibit endocannabinoid biosynthesis by downregulating the mRNA expression of N-acylphosphatidylethanolamine-specific phospholipase D (NAPE-PLD), a key enzyme involved in endocannabinoid biosynthesis [119,120]. Although both classical endocannabinoids (AEA and 2-AG) interact with CB1 and CB2 receptors [121,122], they have different affinities for activation. It is known that, depending on the type of cells, AEA may be a partial or full agonist of CB1 receptors, but it has low affinity for CB2 receptors, for which it is a relatively weak agonist [123,124]. However, 2-AG is a full agonist of both receptors [125], showing greater affinity for CB1 and CB2 receptors than AEA. The microalgae *Chlorococcum amblystomatis* strongly reduces the levels of 2AG and OEA and the expression of all tested receptors. Taking into account changes in the level of endocannabinoids and eicosanoids, it can be concluded that microalgae extracts have anti-inflammatory effects in total. While analyzing the expression of G-protein-related receptors, including CB1, CB2 and PPARγ, which are directly confirmed by the results of the ROS and TNFα levels, it should be emphasized that the regenerative effect of microalgae is largely based on the modification of the expression of these receptors [41,88]. All analyzed receptors are variously involved in metabolic modulations. The activation of CB1 receptors increases the generation of ROS, as well as factors responsible for pro-inflammatory signaling (NF-κB and TNFα), while the activation of CB2 receptors promotes the reduction in oxidative stress and pro-inflammatory signaling by reducing the level of ROS and NF-κB and TNFα [126,127]. Also, the activation of TRPV1 receptors increases the level of ROS [127], while the activation of PPARγ receptors directly enhances the gene expression of proteins with both antioxidant and anti-inflammatory properties [128]. It can also be suggested that the reduction in TNFα levels may also result from the fact that chlorophyll found in microalgae *Chlorococcum amblystomatis* inhibits the expression of the TNF-α gene, which was demonstrated in the case of HEK293 cells induced by bacterial lipopolysaccharides [129]. Analyzing the effectiveness of the antioxidant and anti-inflammatory effects resulting from the significant regeneration of the antioxidant capacity of fibroblasts with a significant inhibition of the generation of pro-inflammatory lipid mediators as a result of increased expression of the CB2 receptor, it can be concluded that the effectiveness of the *N.o.* extract may result from the higher EPA content in this algae strain. This effect is undoubtedly supported by the reduction in the level of eicosanoids and anti-inflammatory endocannabinoids; however, in the case of these parameters, both tested extracts were characterized by moderate effectiveness. Nevertheless, a similar direction of changes can be observed in studies on the regenerative effect of *N.o.* on epidermal cells—keratinocytes [88].

## 5. Limitations

Regardless of the importance of the problem constituting the basis of this study, the presented experiment and the obtained results are subject to certain limitations. The basic one is the solubility and bioavailability of the components of the used lipid extracts in aqueous cell media. Despite the widely used method applying organic solvent, it is not certain that all of the extract compounds are in a physical form that is easily absorbed by cells. Moreover, based on the presented results, it is not able to be determined to what extent lipid compounds are taken by the cells and to what extent they are accumulated in which cellular fraction. However, this would require the use of labeled extract components to check their location in the cell using a fluorescence microscope.

Another limitation of this study is the non-specific pro-oxidant nature of UV radiation. Cells during UV irradiation are in the buffer PBS, which later is exchanged with fresh medium which was not exposed to the pro-oxidant factor. Therefore, it is assumed that all observed pro-oxidant changes come from changes occurring in cells. However, the whole mechanism about what happens in the medium regardless of the metabolism of the experimental cells and to what extent it affects these cells is not known. Therefore, regardless of the comprehensiveness of the conducted research in this study, many aspects of the interactions between the components of lipid extracts and skin cell metabolism require further analysis.

## 6. Conclusions

To sum up, it should be emphasized that lipid extracts from both marine microalgae *Nannochloropsis oceanica* and freshwater microalgae *Chlorococcum amblystomatis* have a protective effect on fibroblasts under physiological conditions and a regenerative effect after exposure to UVA rays, alleviating the disturbed redox balance and pro-inflammatory signaling. It can therefore be suggested that lipid extracts of both *Nannochloropsis oceanica* and *Chlorococcum amblystomatis* may be used as intermediates in the process of creating natural pharmaceuticals/cosmetics for everyday use, as well as those intended to protect dermis cells against the negative effects of UVA radiation. However, their use may be problematic in the case of phototherapy using UVA radiation, e.g., in the case of psoriasis, due to the reduction in the effectiveness of this phototherapy by microalgae. Therefore, this aspect of the effects of microalgae on skin/skin cells requires further research.

## Data Availability

Data is contained within the article and Supplementary Materials.

## Supplementary Materials

The following supporting information can be downloaded at: https://www.mdpi.com/article/10.3390/antiox13030276/s1, Table S1: Phospholipid (PL), glycolipid (GL) and other lipidic components contents in the lipid extracts of *Nannochloropsis oceanica* and *Chlorococcum amblystomatis*; Table S2: Polar lipid composition of the lipid extracts of *Nannochloropsis oceanica*; Table S3: Polar lipid composition of the lipid extracts of *Chlorococcum amblystomatis*.
