# Supplementary material for: Comparison of the Regenerative Metabolic Efficiency of Lipid Extracts from Microalgae Nannochloropsis oceanica and Chlorococcum amblystomatis on Fibroblasts
Source: Antioxidants (Basel). 2024 Feb 24;13(3):276. doi: 10.3390/antiox13030276 (PMC10967502; doi:10.3390/antiox13030276)
Supplement: Supplementary file 1 [file antioxidants-13-00276-s001.zip › Supplementary Table S1.pdf]

Supplementary Table S1

Phospholipid (PL), glycolipid (GL) and other lipidic components contents in the lipid extracts of *Nannochloropsis oceanica* and *Chlorococcum amblystomatis* expressed as mg of lipid per g of lipid extract.

| | Phospholipid content (mg.g-1 of extract) | Glycolipid content (mg.g-1 of extract) | Other components content (neutral lipids, betaine lipids and pigments; mg.g-1 of extract) |
|---|---|---|---|
| *Chlorococcum amblystomatis* | 102.1±14.7 | 674.0±69.9 | 223.9 ± 63.1 |
| *Nannochloropsis oceanica* | 122.6±23.8 | 159.7 ± 16.1 | 717.7 ± 22.4 |
