# Supplementary material for: Comparison of the Regenerative Metabolic Efficiency of Lipid Extracts from Microalgae Nannochloropsis oceanica and Chlorococcum amblystomatis on Fibroblasts
Source: Antioxidants (Basel). 2024 Feb 24;13(3):276. doi: 10.3390/antiox13030276 (PMC10967502; doi:10.3390/antiox13030276)
Supplement: Supplementary file 1 [file antioxidants-13-00276-s001.zip › Supplementary Table S2.pdf]

Supplementary Table S2

Polar lipid composition of the lipid extracts of *Nannochloropsis oceanica* identified by HILIC-ESI-MS and HILIC-ESI-MS/MS in positive ion mode, as [M+H]+ and [M+NH4]+ ions, and in negative ion mode, as [M-H]-. When the fatty acyl chain combination was not possible to assign the lines were filled with "-".

Lipid species (C:N)	Calculated *m/z*	Fatty acyl chains (C:N)
**PC identified as [M+H]+**		
PC(28:1)	676,4917	-
PC(30:3)	700,4917	-
PC(30:1)	704,523	16:1-14:0
PC(31:2)	716,523	16:1-15:1
PC(31:1)	718,5387	16:1-15:0
		17:1-14:0
		15:1-16:0
PC(32:5)	724,4917	-
PC(32:4)	726,5074	16:3-16:1
		16:2/16:2
PC(32:3)	728,523	16:2-16:1
		16:3-16:0
		18:3-14:0
PC(32:2)	730,5387	16:1/16:1
		16:2-16:0
		18:2-14:0
PC(32:1)	732,5543	-
PC(33:3)	742,5387	17:2-16:1
		17:1-16:2
		15:0-18:3
PC(33:2)	744,5543	18:2-15:0
		17:1-16:1
		17:2-16:0
PC(34:7)	748,4917	-
PC(34:6)	750,5074	16:1-18:5
PC(34:5)	752,523	18:3-16:2
		18:4-16:1
		14:0-20:5
		18:2-16:3
PC(34:4)	754,5387	20:4-14:0
		16:1-18:3
		18:4-16:0
		18:2-16:2
		18:1-16:3

Lipid species (C:N)	Calculated m/z	Fatty acyl chains (C:N)
**PC identified as [M+H]+**		
PC(34:3)	756,5543	18:2-16:1
PC(34:2)	758,57	18:1-16:1
		18:2-16:0
PC(34:1)	760,5856	18:1-16:0
		16:1-18:0
PC(35:4)	768,5543	-
PC(35:3)	770,57	-
PC(35:2)	772,5856	-
PC(35:1)	774,6013	16:1-17:0
PC(37:7)	790,5387	17:2-20:5
PC(37:6)	792,5543	21:5-16:1
		17:1-20:5
		17:2-20:4
PC(36:8)	774,5074	-
PC(36:7)	776,523	16:2-20:5
PC(36:6)	778,5387	16:1-20:5
		16:2-20:4
PC(36:5)	780,5543	16:1-20:4
		16:0-20:5
PC(36:4)	782,57	18:2-18:2
		20:3-16:1
		20:4-16:0
PC(36:3)	784,5856	20:3-16:0
		16:1-20:2
		18:2-18:1
PC(36:2)	786,6013	18:1-18:1
		20:2-16:0
		18:2-18:0
		20:1-16:1
PC(38:8)	802,5387	18:3-20:5
PC(38:7)	804,5543	18:2-20:5
		18:3-20:4

Lipid species (C:N)	Calculated m/z	Fatty acyl chains (C:N)
**PC identified as $[M+H]^+$**		
PC(38:6)	806,57	18:2-20:4
		18:1-20:5
		20:3-18:3
PC(38:5)	808,5856	20:4-18:1
PC(38:3)	812,6169	-
PC(38:2)	814,6326	18:2-20:0
PC(38:1)	816,6482	-
PC(40:11)	824,523	-
PC(40:10)	826,5387	20:5/20:5
PC(40:9)	828,5543	20:5-20:4
PC(40:8)	830,57	-
PC(40:5)	836,6169	-

Lipid species (C:N)	Calculated *m/z*	Fatty acyl chains (C:N)
**LPC identified as $[M+H]^+$**		
LPC(14:0)	468,309	14:0
LPC(16:3)	490,2934	16:3
LPC(16:2)	492,309	16:2
LPC(16:1)	494,3247	16:1
LPC(16:0)	496,3403	16:0
LPC(17:0)	510,356	17:0
LPC(17:1)	508,3403	17:1
LPC(17:2)	506,3247	17:2
LPC(18:5)	514,2934	-
LPC(18:4)	516,309	18:4
LPC(18:3)	518,3247	18:3
LPC(18:2)	520,3403	18:2
LPC(18:1)	522,356	18:1
LPC(20:5)	542,3247	20:5
LPC(20:4)	544,3403	20:4
LPC(20:3)	546,356	-
LPC(20:1)	550,3873	-
LPC(20:0)	552,4029	20:0
LPC(22:6)	568,3403	-
LPC(22:5)	570,356	-

Lipid species (C:N)	Calculated *m/z*	Fatty acyl chains (C:N)
**PE identified as $[M+H]^+$**		
PE(30:3)	658,4448	-
PE(30:1)	662,4761	14:0-16:1
		15:1-15:0
PE(30:0)	664,4917	14:0-16:0
		15:0/15:0
PE(32:4)	684,4604	-
PE(32:3)	686,4761	16:2-16:1
PE(32:2)	688,4917	16:1/16:1
		16:2-16:0
PE(32:1)	690,5074	-
PE(34:6)	708,4604	-
PE(34:5)	710,4761	-
PE(34:4)	712,4917	18:3-16:1
		14:0-20:4
PE(34:3)	714,5074	18:2-16:1
		20:3-14:0
PE(34:2)	716,523	16:1-18:1
PE(36:8)	732,4604	-
PE(36:7)	734,4761	-
PE(36:6)	736,4917	16:1-20:5
		16:2-20:4
PE(36:5)	738,5074	16:1-20:4
PE(36:4)	740,523	20:3-16:1
		20:4-16:0
PE(36:3)	742,5387	-
PE(36:2)	744,5543	18:1/18:1
PE(38:9)	758,4761	-
PE(38:8)	760,4917	18:4-20:4
PE(38:7)	762,5074	18:3-20:4
		20:3-18:4
		18:2-20:5
PE(38:6)	764,523	18:2-20:4
PE(38:5)	766,5387	18:1-20:4
PE(40:10)	784,4917	20:5/20:5
PE(40:9)	786,5074	20:4-20:5
PE(40:8)	788,523	20:4-20:4
PE(40:7)	790,5387	20:3-20:4
		20:5-20:2

Lipid species (C:N)	Calculated *m/z*	Fatty acyl chains (C:N)
**LPE identified as [M+H]⁺**		
LPE(14:0)	426,2621	14:0
LPE(16:3)	448,2464	-
LPE(16:2)	450,2621	16:2
LPE(16:1)	452,2777	16:1
LPE(16:0)	454,2934	-
LPE(18:4)	474,2621	18:4
LPE(18:3)	476,2777	18:3
LPE(18:2)	478,2934	18:2
LPE(18:1)	480,309	18:1
LPE(20:5)	500,2777	20:5
LPE(20:4)	502,2934	20:4
LPE(20:3)	504,309	-
LPE(20:2)	506,3247	-

Lipid species (C:N)	Calculated *m/z*	Fatty acyl chains (C:N)
**PG identified as [M−H]⁻**		
PG(30:1)	691,455	14:0-16:1
PG(31:0)	707,4863	15:0-16:0
PG(31:1)	705,4707	15:0-16:1
		15:1-16:0
PG(32:1)	719,4863	16:0-16:1
		14:0-18:1
PG(32:2)	717,4707	16:1/16:1
		16:0-16:2
PG(33:1)	733,502	16:1-17:0
PG(34:1)	747,5176	16:0-18:1
PG(34:2)	745,502	16:0-18:2
		16:1-18:1
PG(34:5)	739,455	14:0-20:5
PG(35:5)	753,4707	15:0-20:5
PG(36:2)	773,5333	-
PG(36:5)	767,4863	16:0-20:5
		16:1-20:4
PG(36:5(OH))	783,4812	(16:0-OH)-20:5
PG(36:6)	765,4707	20:5-16:1

Lipid species (C:N)	Calculated *m/z*	Fatty acyl chains (C:N)
**LPG identified as [M−H]⁻**		
LPG(16:0)	483,2723	16:0

Lipid species (C:N)	Calculated *m/z*	Fatty acyl chains (C:N)
**PI identified as [M−H]⁻**		
PI(28:0)	753,4554	14:0/14:0
PI(30:1)	779,4711	16:1-14:0
PI(32:1)	807,5024	16:1-16:0
	807,5024	18:1-14:0
PI(32:2)	805,4867	16:1/16:1
	805,4867	16:0-16:2
	805,4867	18:2-14:0
PI(33:1)	821,518	16:1-17:0
	821,518	16:0-17:1
PI(34:1)	835,5337	18:1-16:0
	835,5337	16:1-18:0
PI(34:2)	833,518	18:2-16:0
	833,518	16:1-18:1
PI(34:3)	831,5024	18:2-16:1
	831,5024	16:2-18:1
PI(34:5)	827,4711	14:0-20:5
PI(36:6)	853,4867	20:5-16:1
PI(40:10)	901,4867	20:5/20:5

Lipid species (C:N)	Calculated *m/z*	Fatty acyl chains (C:N)
**MGTS identified as [M+H]⁺**		
MGTS(14:1)	444,3325	14:1
MGTS(14:0)	446,3482	14:0
MGTS(15:0)	460,3638	15:0
MGTS(16:4)	466,3169	16:4
MGTS(16:3)	468,3325	16:3
MGTS(16:2)	470,3482	-
MGTS(16:1)	472,3638	16:1
MGTS(16:0)	474,3795	-
MGTS (17:1)	486,3795	17:1
MGTS (17:2)	484,3638	17:2
MGTS(18:5)	492,3325	18:5
MGTS(18:4)	494,3482	18:4
MGTS(18:3)	496,3638	18:3
MGTS(18:2)	498,3795	18:2
MGTS(18:1)	500,3951	18:1
MGTS(20:5)	520,3638	20:5
MGTS(20:4)	522,3795	-

Lipid species (C:N)	Calculated *m/z*	Fatty acyl chains (C:N)
**DGTS identified as [M+H]$^+$**		
DGTS(28:1)	654,5309	16:1-12:0
		14:1-14:0
DGTS(28:0)	656,5465	14:0/14:0
		12:0-16:0
DGTS(30:3)	678,5309	16:3-.14:0
DGTS(30:2)	680,5465	16:2-14:0
		16:1-14:1
DGTS(30:1)	682,5622	16:1-14:0
DGTS(32:5)	702,5309	20:5-12:0
DGTS(32:4)	704,5465	18:4-14:0
DGTS(32:3)	706,5622	-
DGTS(32:2)	708,5778	16:1/16:1
		18:2-14:0
		16:2-16:0
DGTS(32:1)	710,5935	16:1-16:0
		18:1-14:0
DGTS(33:2)	722,5935	17:1-16:1
		18:2-15:0
		17:2-16:0
DGTS(34:6)	728,5465	20:5-14:1
DGTS(34:5)	730,5622	20:5-14:0
DGTS(34:4)	732,5778	20:4-14:0
DGTS(34:3)	734,5935	20:3-14:0
		16:1-18:2
		18:3-16:0
DGTS(34:2)	736,6091	18:2-16:0
		18:1-16:1
		20:2-14:0
DGTS(34:1)	738,6248	20:1-14:0
		16:0-18:1
DGTS(36:7)	754,5622	16:2-20:5
DGTS(36:6)	756,5778	16:1-20:5
DGTS(36:5)	758,5935	20:5-16:0
DGTS(36:4)	760,6091	-
DGTS(36:3)	762,6248	20:3-16:0
		20:2-16:1
		18:2-18:1
DGTS(36:2)	764,6404	-
DGTS (37:5)	772,6091	20:5-17:0
		20:4-17:1
DGTS(38:10)	776,5465	18:5-20:5
DGTS(38:9)	778,5622	18:4-20:5
DGTS(38:8)	780,5778	-

Lipid species (C:N)	Calculated *m/z*	Fatty acyl chains (C:N)
**DGTS identified as [M+H]$^+$**		
DGTS(38:7)	782,5935	20:5-18:2
		18:3-20:4
DGTS(38:6)	784,6091	-
DGTS(38:5)	786,6248	20:4-18:1
		20:5-18:0
DGTS(40:10)	804,5778	20:5/20:5
DGTS(40:9)	806,5935	-

Lipid species (C:N)	Calculated *m/z*	Fatty acyl chains (C:N)
**SQDG identified as [M−H]$^-$**		
SQDG(28:0)	737,451	14:0/14:0
SQDG(30:0)	765,4823	16:0-14:0
SQDG(30:1)	763,4666	16:1-14:0
SQDG(31:1)	777,4823	15:0-16:1
SQDG(32:1)	791,4979	-
SQDG(32:2)	789,4823	16:1/16:1
SQDG(32:3)	787,4666	-
SQDG(34:1)	819,5292	16:0-18:1
SQDG(34:2)	817,5136	16:0-18:2
SQDG(36:5)	839,4979	-

Lipid species (C:N)	Calculated m/z	Fatty acyl chains (C:N)
**MGDG identified as [M+NH$_4$]$^+$**		
MGDG(30:1)	718,5464	-
MGDG(32:5)	738,5156	-
MGDG(32:2)	744,5626	-
MGDG(32:1)	746,5777	-
MGDG(34:5)	766,5469	14:0-20:5
		16:1-18:4
MGDG(34:2)	772,5933	-
MGDG(34:1)	774,609	-
MGDG(36:6)	792,5625	16:1-20:5
MGDG(36:5)	794,5782	-
MGDG(38:7)	818,5782	-
MGDG(40:10)	840,5626	-
MGDG(40:9)	842,5782	-
MGDG(40:8)	844,5939	-

Lipid species (C:N)	Calculated *m/z*	Fatty acyl chains (C:N)
**DGDG identified as $[M+NH_4]^+$**		
DGDG(30:1)	880,5997	14:0-16:1
DGDG(30:0)	882,6154	-
DGDG(32:5)	900,5684	-
DGDG(32:3)	904,5997	-
DGDG(32:2)	906,6154	16:1/16:1
		14:0-18:2
		16:0-16:2
DGDG(32:1)	908,631	16:0-16:1
		-
DGDG(34:5)	928,5997	14:0-20:5
DGDG(34:3)	932,631	-
DGDG(34:2)	934,6467	-
DGDG(34:1)	936,6623	-
DGDG(35:5)	942,6154	15:0-20:5
DGDG(36:7)	952,5997	-
DGDG(36:6)	954,6154	16:1-20:5
DGDG(36:5)	956,631	-
DGDG(38:7)	980,631	18:2-20:5
DGDG(38:6)	982,6467	-
DGDG(40:10)	1002,6154	20:5/20:5

Lipid species (C:N)	Calculated *m/z*	Fatty acyl chains (C:N)
**MGMG identified as $[M+NH_4]^+$**		
MGMG(16:0)	510,3642	-

Lipid species (C:N)	Calculated *m/z*	Fatty acyl chains (C:N)
**DGMG identified as $[M+NH_4]^+$**		
DGMG(14:0)	644,3857	-
DGMG(16:1)	670,4014	-
DGMG(16:0)	672,417	16:0
DGMG(20:5)	718,4014	-

Lipid species (C:N)	Calculated *m/z*	Fatty acyl chains (C:N)
**Cer identified as $[M+H]^+$**		
Cer(d32:2)	508,473	d18:1/14:1
Cer(d32:1)	510,4886	-

Lipid species (C:N)	Calculated *m/z*	Fatty acyl chains (C:N)
**PI-Cer identified as $[M-H]^-$**		
PI-Cer(d18:1/14:0)	750,4921	d18:1/14:0
PI-Cer(d18:1/14:1)	748,4765	d18:1/14:1

Abbreviations:
PC, phosphatidylcholine;
PE, phosphatidylethanolamine;
PG, phosphatidylglycerol;
PI, phosphatidylinositol;
LPC, lysophosphatidylcholine;
LPE, lysophosphatidylethanolamine;
LPG, lysophosphatidylglycerol;
MGTS, monoacylglyceryl 3-O-4´-(N,N,N-trimethyl) homoserine;
DGTS, diacylglyceryl 3-O-4´-(N,N,N-trimethyl) homoserine;
SQDG, sulfoquinovosyldiacylglycerol;
MGDG, monogalactosyldiacylglycerol;
DGDG, digalactosyldiacylglycerol;
MGMG, monogalactosylmonoacylglycerol;
DGMG, digalactosylmonoacylglycerol;
Cer, ceramide;
PI-Cer, inositolphosphoceramide.
