# Supplementary material for: Comparison of the Regenerative Metabolic Efficiency of Lipid Extracts from Microalgae Nannochloropsis oceanica and Chlorococcum amblystomatis on Fibroblasts
Source: Antioxidants (Basel). 2024 Feb 24;13(3):276. doi: 10.3390/antiox13030276 (PMC10967502; doi:10.3390/antiox13030276)
Supplement: Supplementary file 1 [file antioxidants-13-00276-s001.zip › Supplementary Table S3.pdf]

Supplementary Table S3

Polar lipid composition of the lipid extracts of *Chlorococcum amblystomatis* identified by HILIC-ESI-MS and HILIC-ESI-MS/MS in positive ion mode, as [M+H]+ and [M+NH4]+ ions, and in negative ion mode, as [M-H]-. When the fatty acyl chain combination was not possible to assign the lines were filled with "-".

| Lipid species (C:N) | Calculated m/z | Fatty acyl chains (C:N) |
|---|---|---|
| **PC identified as [M+H]+** | | |
| PC(28:1) | 676,4917 | - |
| PC(30:3) | 700,4917 | - |
| PC(30:1) | 704,523 | 16:1-14:0 |
| PC(30:0) | 706,5387 | - |
| PC(32:1) | 732,5543 | 16:0-16:1 |
| PC(32:2) | 730,5387 | 16:1-16:1 |
| PC(32:3) | 728,523 | 16:1-16:2 |
| PC(32:4) | 726,5074 | - |
| PC(32:5) | 724,4917 | - |
| PC(34:1) | 760,5856 | 16:0-18:1 |
| | | 16:1-18:0 |
| PC(34:2) | 758,57 | 16:1-18:1 |
| | | 16:0-18:2 |
| PC(34:3) | 756,5543 | 16:1-18:2 |
| PC(34:4) | 754,5387 | 16:1-18:3 |
| | | 16:2-18:2 |
| PC(34:5) | 752,523 | - |
| PC(34:6) | 750,5074 | - |
| PC(34:7) | 748,4917 | - |
| PC(36:2) | 786,6013 | - |
| PC(36:3) | 784,5856 | 18:1-18:2 |
| PC(36:4) | 782,57 | 18:2-18:2 |
| | | 16:0-20:4 |
| PC(36:5) | 780,5543 | 18:2-18:3 |
| | | 16:0-20:5 |
| | | 16:1-20:4 |
| PC(36:6) | 778,5387 | 16:1-20:5 |
| PC(36:7) | 776,523 | - |
| PC(38:5) | 808,5856 | 18:1-20:4 |
| PC(38:6) | 806,57 | 18:2-20:4 |
| | | 18:1-20:5 |
| PC(38:7) | 804,5543 | 18:2-20:5 |
| PC(38:8) | 802,5387 | - |
| PC(38:9) | 800,523 | - |
| PC(40:10) | 826,5387 | - |
| PC(40:5) | 836,6169 | - |
| PC(40:7) | 832,5856 | - |
| PC(40:8) | 830,57 | - |
| PC(40:9) | 828,5543 | - |

| Lipid species (C:N) | Calculated m/z | Fatty acyl chains (C:N) |
|---|---|---|
| **LPC identified as [M+H]+** | | |
| LPC14:0) | 468,309 | - |
| LPC(16:0) | 496,3403 | - |
| LPC(16:1) | 494,3247 | 16:1 |
| LPC(16:2) | 492,309 | - |
| LPC(18:1) | 522,356 | 18:1 |
| LPC(18:2) | 520,3403 | 18:2 |
| LPC(18:3) | 518,3247 | - |
| LPC(18:4) | 516,309 | - |
| LPC(20:3) | 546,356 | - |
| LPC(20:4) | 544,3403 | - |
| LPC(20:5) | 542,3247 | - |

| Lipid species (C:N) | Calculated *m/z* | Fatty acyl chains (C:N) |
|---|---|---|
| **PE identified as [M+H]+ - polar head as neutral loss** | | |
| PE(30:1) | 662,4761 | 14:0-16:1 |
| PE(30:3) | 658,4448 | - |
| PE(32:1) | 690,5074 | - |
| PE(32:2) | 688,4917 | 16:1-16:1 |
| PE(32:4) | 684,4604 | 16:0-16:4 |
| PE(34:1) | 718,5387 | 16:0-18:1 |
| PE(34:2) | 716,523 | 16:1-18:1 |
| | | 16:0-18:2 |
| PE(34:3) | 714,5074 | - |
| PE(34:4) | 712,4917 | 16:0-18:4 |
| PE(34:5) | 710,4761 | 16:1-18:4 |
| PE(36:2) | 744,5543 | 18:1-18:1 |
| PE(36:5) | 738,5074 | 18:1-18:4 |
| PE(36:6) | 736,4917 | - |

| Lipid species (C:N) | Calculated m/z | Fatty acyl chains (C:N) |
|---|---|---|
| **LPE identified as [M+H]+ - polar head as neutral loss** | | |
| LPE(14:0) | 426,2621 | - |
| LPE(16:0) | 454,2934 | 16:0 |
| LPE(16:1) | 452,2777 | - |
| LPE(16:4) | 446,2308 | - |
| LPE(18:1) | 480,309 | 18:1 |
| LPE(18:2) | 478,2934 | - |
| LPE(18:3) | 476,2777 | - |
| LPE(18:4) | 474,2621 | 18:4 |
| LPE(20:5) | 500,2777 | - |

| Lipid species (C:N) | Calculated m/z | Fatty acyl chains (C:N) |
|---|---|---|
| **PG identified as [M–H]−** | | |
| PG(30:0) | 693,4707 | 14:0-16:0 |
| PG(30:1) | 691,455 | - |
| PG(32:0) | 721,502 | - |
| PG(32:1) | 719,4863 | 16:1-16:0 |
| | | 14:0-18:1 |
| PG(32:2) | 717,4707 | 16:1-16:1 |
| PG(34:1) | 747,5176 | 16:0-18:1 |
| PG(34:2) | 745,502 | - |
| PG(34:3) | 743,4867 | 16:0-18:3 |
| PG(34:4) | 741,4707 | 16:0-18:4 |
| | | 16:1-18:3 |
| PG(34:5) | 739,455 | 14:0-20:5 |
| | | 16:2-18:3 |
| PG(36:2) | 773,5333 | 18:1-18:1 |
| PG(36:5) | 767,4864 | 16:0-20:5 |
| PG(36:6) | 765,4707 | 16:1-20:5 |
| PG(38:5) | 795,5176 | - |
| PG(32:2-OH) | 733,4656 | (16:0-OH)-16:2 |
| PG(34:1-OH) | 763,5125 | (18:0-OH)-16:1 |
| PG(34:2-OH) | 761,4969 | (18:0-OH)-16:2 |
| PG(34:3-OH) | 759,4812 | (18:3-OH)-16:0 |
| | | (18:2-OH)-16:1 |
| | | (18:1-OH)-16:2 |
| | | (18:0-OH)-16:3 |
| PG(34:4-OH) | 757,4656 | (18:4-OH)-16:0 |
| | | (18:3-OH)-16:1 |
| | | (18:2-OH)-16:2 |
| PG(34:5-OH) | 755,4499 | (18:3-OH)-16:2 |
| | | (18:4-OH)-16:1 |
| PG(36:5-OH) | 783,4812 | (16:0-OH)-20:5 |
| | | (20:5-OH)-16:0 |
| PG(36:6-OH) | 781,4656 | (20:5-OH)-16:1 |

| Lipid species (C:N) | Calculated *m/z* | Fatty acyl chains (C:N) |
|---|---|---|
| **PI identified as [M–H]−** | | |
| PI(32:1) | 807,5024 | 16:0-16:1 |
| PI(34:1) | 835,5318 | 16:0-18:1 |
| PI(32:1) | 807,5024 | 16:0-16:1 |

| Lipid species (C:N) | Calculated m/z | Fatty acyl chains (C:N) |
|---|---|---|
| **MGTS identified as [M+H]+** | | |
| MGTS(14:0) | 446,3482 | 14:0 |
| MGTS(14:1) | 444,3325 | 14:1 |
| MGTS(16:0) | 474,3795 | 16:0 |
| MGTS(16:1) | 472,3638 | 16:1 |
| MGTS(16:2) | 470,3482 | 16:2 |
| MGTS(16:3) | 468,3325 | - |
| MGTS(16:4) | 466,3169 | 16:4 |
| MGTS(18:0) | 502,4108 | 18:0 |
| MGTS(18:1) | 500,3951 | 18:1 |
| MGTS(18:2) | 498,3795 | - |
| MGTS(18:3) | 496,3638 | - |
| MGTS(18:4) | 494,3482 | 18:4 |
| MGTS(18:5) | 492,3325 | - |
| MGTS(20:0) | 530,4421 | 20:0 |
| MGTS(20:4) | 522,3795 | - |
| MGTS(20:5) | 520,3638 | 20:5 |

| Lipid species (C:N) | Calculated m/z | Fatty acyl chains (C:N) |
|---|---|---|
| **DGTS identified as $[M+H]^+$** | | |
| DGTS(28:1) | 654,5309 | - |
| DGTS(30:0) | 684,5778 | 16:0-14:0 |
| DGTS(30:1) | 682,5622 | 16:1-16:0 |
| DGTS(30:2) | 680,5465 | 16:2-14:0 |
| DGTS(30:3) | 678,5309 | - |
| DGTS(30:4) | 676,5152 | 16:4-16:0 |
| DGTS(30:5) | 674,4996 | 16:4-14:1 |
| DGTS(32:0) | 712,6091 | 16:0-16:0 |
| DGTS(32:1) | 710,5935 | 16:1-16:0 |
| DGTS(32:2) | 708,5778 | 16:0-16:2 |
| | | 16:1-16:1 |
| DGTS(32:3) | 706,5622 | 16:3-16:0 |
| DGTS(32:4) | 704,5465 | 14:0-18:4 |
| | | 16:4-16:0 |
| | | 16:3-16:1 |
| DGTS(32:5) | 702,5309 | - |
| DGTS(32:6) | 700,5152 | 16:4-16:2 |
| | | 16:3-16:3 |
| DGTS(32:7) | 698,4996 | 16:4-16:3 |
| DGTS(32:8) | 696,4839 | 16:4-16:4 |
| DGTS(34:1) | 738,6248 | 16:0-18:1 |
| DGTS(34:2) | 736,6091 | 16:1-18:1 |
| | | 18:2-16:0 |
| | | 20:2-14:0 |

| Lipid species (C:N) | Calculated m/z | Fatty acyl chains (C:N) |
|---|---|---|
| **DGTS identified as $[M+H]^+$** | | |
| DGTS(34:3) | 734,5935 | 16:2-18:1 |
| | | 16:1-18:2 |
| | | 20:3-14:0 |
| DGTS(34:4) | 732,5778 | 18:4-16:0 |
| | | 18:3-16:1 |
| DGTS(34:5) | 730,5622 | 18:4-16:1 |
| | | 18:1-16:4 |
| | | 18:2-16:3 |
| DGTS(34:5-OH) | 746,5571 | (16:4-OH)-18:1 |
| | | (16:1-OH)-18:4 |
| | | (16:0-OH)-18:5 |
| DGTS(34:6) | 728,5465 | - |
| DGTS(34:7) | 726,5309 | 16:4-18:3 |
| | | 16:3-18:4 |
| DGTS(34:7-OH) | 742,5258 | (16:4-OH)-18:3 |
| | | (16:3-OH)-18:4 |
| DGTS(34:8) | 724,5152 | 16:4-18:4 |
| DGTS(36:10) | 748,5152 | 18:5-18:5 |
| DGTS(36:4) | 760,6091 | 16:0-20:4 |
| | | 18:3-18:1 |
| | | 18:2-18:2 |
| | | 20:3-16:1 |
| | | 20:2-16:2 |
| | | 20:1-16:3 |
| | | 20:0-16:4 |
| DGTS(36:5) | 758,5935 | 18:4-18:1 |
| | | 20:5-16:0 |
| | | 20:4-16:1 |
| | | 20:3-16:2 |
| | | 20:2-16:3 |
| | | 20:1-16:4 |
| | | 16:1-20:4 |
| DGTS(36:6) | 756,5778 | 16:1-20:5 |
| | | 20:3-16:3 |
| | | 18:2-18:4 |
| | | 18:3-18:3 |
| DGTS(36:7) | 754,5622 | 18:4-18:3 |
| DGTS(36:8) | 752,5465 | 18:4-18:4 |
| DGTS(36:9) | 750,5309 | 18:5-18.4 |
| DGTS(38:10) | 776,5465 | 20:5-18:5 |
| DGTS(38:7) | 782,5935 | 20:5-18:2 |
| DGTS(38:8) | 780,5778 | 20:5-18:3 |
| DGTS(40:10) | 804,5778 | 20:5-20:5 |
| DGTS(40:9) | 806,5935 | 20:4-20:5 |

| Lipid species (C:N) | Calculated m/z | Fatty acyl chains (C:N) |
|---|---|---|
| **SQDG identified as $[M-H]^-$** | | |
| SQDG(28:0) | 737,451 | - |
| SQDG(30:0) | 765,4823 | 16:0-14:0 |
| SQDG(30:1) | 763,4666 | 16:1-14:0 |
| SQDG(32:0) | 793,5136 | - |
| SQDG(32:1) | 791,4979 | 16:0-16:1 |
| SQDG(32:2) | 789,4823 | 16:1-16:1 |
| | | 16:0-16:2 |
| SQDG(32:3) | 787,4666 | 16:1-16:2 |
| | | 16:0-16:3 |
| | | 18:1-14:1 |
| SQDG(32:4) | 785,451 | 16:0-16:4 |
| | | 16:1-16:3 |
| | | 16:2-16:2 |
| SQDG(34:0) | 821,5449 | 16:0-18:0 |
| SQDG(34:1) | 819,5292 | 16:0-18:1 |
| SQDG(34:3) | 815,4979 | 16:0-18:3 |
| | | 18:1-16:2 |
| SQDG(34:4) | 813,4823 | 16:0-18:4 |
| | | 16:1-18:3 |
| | | 16:2-18:2 |
| | | 18:1-16:3 |
| | | 20:4-14:0 |
| SQDG(36:0) | 849,5762 | 16:0-20:0 |
| SQDG(36:3) | 843,5292 | - |
| SQDG(36:4) | 841,5136 | 16:0-20:4 |
| SQDG(36:5) | 839,4979 | - |
| SQDG(36:6) | 837,4823 | 16:1-20:5 |
| | | 20:4-16:2 |
| | | 18:2-18:4 |
| | | 18:3-18:3 |
| SQDG(36:7) | 835,4666 | 18:3-18:4 |
| | | 20:4-16:3 |
| | | 20:5-16:2 |
| SQDG(34:3-OH) | 831,4928 | - |
| SQDG(34:4-OH) | 829,4772 | - |

| Lipid species (C:N) | Calculated *m/z* | Fatty acyl chains (C:N) |
|---|---|---|
| **MGDG identified as $[M+NH_4]^+$** | | |
| MGDG(30:0) | 720,5625 | - |
| MGDG(30:1) | 718,5464 | 16:1-14:0 |
| MGDG(32:0) | 748,5939 | 16:0-16:0 |
| MGDG(32:1) | 746,5777 | 16:1-16:0 |
| MGDG(32:2) | 744,5626 | 16:1-16:1 |
| | | 14:0-18:2 |
| | | 16:0-16:2 |
| MGDG(32:3) | 742,5464 | - |
| MGDG(32:4) | 740,5307 | - |
| MGDG(32:5) | 738,5156 | - |
| MGDG(32:6) | 736,4994 | - |
| MGDG(32:7) | 734,4843 | - |
| MGDG(32:8) | 732,4687 | 16:4-16:4 |
| MGDG(34:1) | 774,609 | - |
| MGDG(34:2) | 772,5933 | 18:2-16:0 |
| MGDG(34:3) | 770,5782 | 18:3-16:0 |
| | | 18:2-16:1 |
| MGDG(34:4) | 768,5626 | 16:2-18:2 |
| | | 16:1-18:3 |
| | | 16:0-18:4 |
| | | 16:3-18:1 |
| | | 16:4-18:0 |
| MGDG(34:5) | 766,5469 | - |
| MGDG(34:7) | 762,5156 | 16:4-18:3 |
| | | 18:4-16:3 |
| MGDG(34:8) | 760,5 | - |
| MGDG(36:3) | 798,6095 | - |
| MGDG(36:4) | 796,5933 | - |
| MGDG(36:5) | 794,5782 | 18:3-18:2 |
| | | 18:1-18:4 |
| | | 20:5-16:0 |
| | | 16:4-20:2 |
| | | 16:1-20:4 |
| MGDG(36:6) | 792,5625 | 18:3-18:3 |
| | | 16:1-20:5 |
| | | 16:4-20:2 |
| MGDG(36:7) | 790,5469 | - |
| MGDG(36:8) | 788,5313 | - |
| MGDG(38:6) | 820,5939 | 18:1-20:5 |
| | | 18:2-20:4 |
| | | 18:3-20:3 |
| MGDG(38:7) | 818,5782 | 20:5-18:2 |
| | | 18:3-20:4 |
| MGDG(38:8) | 816,5626 | - |
| MGDG(40:10) | 840,5626 | 20:5-20:5 |
| MGDG(40:8) | 844,5939 | 20:4-20:4 |
| MGDG(40:7) | 846,6095 | - |

| Lipid species (C:N) | Calculated m/z | Fatty acyl chains (C:N) |
|---|---|---|
| **DGDG identified as [M+NH4]+** | | |
| DGDG(30:0) | 882,6154 | - |
| DGDG(30:1) | 880,5997 | 16:1-14:0 |
| DGDG(32:1) | 908,631 | 16:0-16:1 |
| DGDG(32:2) | 906,6154 | 16:0-16:2 |
| | | 16:1-16:1 |
| DGDG(32:3) | 904,5997 | 18:3-14:0 |
| | | 16:0-16:3 |
| | | 16:1-16:2 |
| DGDG(32:4) | 902,5841 | 18:3-14:1 |
| | | 16:0-16:4 |
| | | 14:0-18:4 |
| DGDG(32:5) | 900,5684 | 16:1-16:4 |
| DGDG(32:6) | 898,5528 | - |
| DGDG(34:1) | 936,6623 | 18:1-16:0 |
| DGDG(34:2) | 934,6467 | 18:1-16:1 |
| | | 18:2-16:0 |
| DGDG(34:3) | 932,631 | 16:0-18:3 |
| | | 18:1-16:2 |
| DGDG(34:4) | 930,6154 | 18:1-16:3 |
| | | 18:2-16:2 |
| | | 18:3-16:1 |
| | | 18:4-16:0 |
| | | 14:0-20:4 |
| DGDG(34:5) | 928,5997 | 18:3-16:2 |
| | | 18:2-16:3 |
| | | 18:1-16:4 |
| | | 16:1-18:4 |
| | | 14:0-20:5 |
| DGDG(34:6) | 926,5841 | - |
| DGDG(34:7) | 924,5684 | 18:3-16:4 |
| | | 18:4-16:3 |
| DGDG(34:8) | 922,5528 | - |
| DGDG(36:2) | 962,678 | - |
| DGDG(36:3) | 960,6623 | 18:3-18:0 |
| | | 18:1-18:2 |
| DGDG(36:4) | 958,6467 | - |
| DGDG(36:5) | 956,631 | 20:5-16:0 |
| | | 18:1-18:4 |
| | | 20:1-16:4 |
| DGDG(36:6) | 954,6154 | 18:3-18:3 |
| | | 20:5-16:1 |
| | | 18:4-18:2 |
| DGDG(36:7) | 952,5997 | 18:3-18:4 |
| DGDG(36:8) | 950,5841 | 18:4-18:4 |
| DGDG(38:6) | 982,6467 | - |
| DGDG(38:7) | 980,631 | 18:2-20:5 |
| DGDG(40:10) | 1002,6154 | 20:5-20:5 |
| DGDG(40:9) | 1004,631 | - |

| Lipid species (C:N) | Calculated m/z | Fatty acyl chains (C:N) |
|---|---|---|
| **MGMG identified as [M+NH4]+** | | |
| MGMG(14:0) | 482,3329 | 14:0 |
| MGMG(16:0) | 510,3642 | 16:0 |
| MGMG(16:1) | 508,3486 | 16:1 |
| MGMG(16:3) | 504,3173 | ** |
| MGMG(16:4) | 502,3016 | 16:4 |
| MGMG(16:4-OH) | 518,2965 | 16:4-OH |
| MGMG(18:1) | 536,3799 | 18:1 |
| MGMG(18:3) | 532,3486 | 18:3 |
| MGMG(18:4) | 530,3329 | - |
| MGMG(20:4) | 558,3642 | - |
| MGMG(20:5) | 556,3486 | - |

| Lipid species (C:N) | Calculated m/z | Fatty acyl chains (C:N) |
|---|---|---|
| **DGMG identified as [M+NH4]+** | | |
| DGMG(14:0) | 644,3857 | - |
| DGMG(16:0) | 672,417 | 16:0 |
| DGMG(16:1) | 670,4014 | 16:1 |
| DGMG(16:2) | 668,3857 | - |
| DGMG(16:3) | 666,3701 | - |
| DGMG(16:4) | 664,3544 | - |
| DGMG(18:1) | 698,4327 | 18:1 |
| DGMG(18:2) | 696,417 | - |
| DGMG(18:3) | 694,4014 | 18:3 |
| DGMG(18:4) | 692,3857 | - |
| DGMG(20:5) | 718,4014 | - |
| DGMG(14:0) | 644,3857 | - |

| Lipid species (C:N) | Calculated m/z | Fatty acyl chains (C:N) |
|---|---|---|
| **SQMG identified as [M–H]−** | | |
| SQMG(16:0) | 555,2839 | - |

| Lipid species (C:N) | Calculated m/z | Fatty acyl chains (C:N) |
|---|---|---|
| **PI-Cer identified as [M–H]−** | | |
| PI-Cer(d18:1/14:0) | 750,4921 | - |

***Abbreviations:***
**PC**, phosphatidylcholine;
**PE**, phosphatidylethanolamine;
**PG**, phosphatidylglycerol;
**PI**, phosphatidylinositol;
**LPC**, lysophosphatidylcholine;
**LPE**, lysophosphatidylethanolamine;
**LPG**, lysophosphatidylglycerol;
**MGTS**, monoacylglyceryl 3-O-4´-(N,N,N-trimethyl) homoserine;
**DGTS**, diacylglyceryl 3-O-4´-(N,N,N-trimethyl) homoserine;
**SQDG**, sulfoquinovosyldiacylglycerol;
**MGDG**, monogalactosyldiacylglycerol;
**DGDG**, digalactosyldiacylglycerol;
**MGMG**, monogalactosylmonoacylglycerol;
**DGMG**, digalactosylmonoacylglycerol;
**SQMG**, sulfoquinovosylmonoacylglycerol;
**PI-Cer**, inositolphosphoceramide.
